# CGRP-dependent molecular signaling drives bone marrow stem cell osteogenesis in distraction osteogenesis

**DOI:** 10.3389/fbioe.2025.1641476

**Published:** 2025-09-24

**Authors:** Yimurang Hamiti, Kai Liu, Sulong Wang, Xin Yang, Xiriaili Kadier, Aihemaitijiang Yusufu

**Affiliations:** ^1^ Department of Trauma and Microreconstructive Surgery, The First Affiliated Hospital of Xinjiang Medical University, Urumqi, Xinjiang, China; ^2^ Xinjiang Key Laboratory of Trauma Repair and Reconstruction, Urumqi, Xinjiang, China

**Keywords:** CGRP, bone marrow mesenchymal stem cells, distraction osteogenesis, cAMP/PKA/CREB pathway, osteogenic differentiation, bone regeneration

## Abstract

Distraction osteogenesis (DO) represents a promising approach for treating large bone defects, yet prolonged consolidation periods limit clinical efficacy, and the molecular mechanisms underlying neuropeptide regulation of bone regeneration remain poorly understood. We systematically investigated calcitonin gene-related peptide (CGRP)-dependent molecular signaling effects on rat bone marrow mesenchymal stem cells (BMSCs) through comprehensive *in vitro* analyses and evaluated therapeutic efficacy in a rat femoral DO model (n = 108). CGRP (10^−8^ M) significantly enhanced BMSC proliferation, migration, and osteogenic differentiation while reducing apoptosis by 47%. Molecular analysis revealed rapid cyclic adenosine monophosphate (cAMP) elevation (2.3-fold within 30 min) and subsequent PKA/CREB pathway activation, upregulating key osteogenic genes (*Runx2*, *Sp7*, *Spp1*, and *Bglap*). The CGRP receptor antagonist and the PKA inhibitor completely abolished these effects, confirming pathway dependence. *In vivo*, local CGRP treatment accelerated bone formation, improving bone mineral density (1.8-fold), trabecular microarchitecture, and biomechanical properties, including ultimate load (89% increase) and stiffness (76% increase), compared to controls. These findings demonstrate that CGRP-dependent molecular signaling drives bone marrow stem cell osteogenesis through cAMP/PKA/CREB activation, providing mechanistic insights into neuropeptide regulation of bone regeneration and identifying CGRP as a promising therapeutic target for optimizing distraction osteogenesis outcomes.

## 1 Introduction

Large bone defects represent one of the most challenging problems in orthopedic surgery, with diverse etiologies including severe trauma, tumor resection, infection, and congenital malformations (Verrier et al., 2016; Yu et al., 2025). Although autologous bone grafting is considered the gold standard, its inherent limitations significantly restrict widespread application, including limited donor bone availability, donor site complications (pain, infection, and nerve injury), and prolonged surgical time (Myeroff and Archdeacon, 2011; Attia et al., 2022; Farrokhi et al., 2022). Therefore, developing novel bone regeneration strategies has become a research hotspot in orthopedic medicine.

Distraction osteogenesis (DO), as an endogenous tissue engineering strategy, effectively stimulates osteogenic potential through controlled mechanical traction applied to osteotomy ends (Amir et al., 2009; Sailhan, 2011). DO has been widely applied in long bone lengthening, deformity correction, nonunion treatment, and large bone defect reconstruction (Gaida et al., 2021; Kummer et al., 2024; Feng et al., 2016). However, DO treatment typically requires prolonged consolidation periods, with complications such as nonunion, delayed healing, and refracture imposing physiological and psychological burdens on patients (Schmidmaier and Moghaddam, 2015; Niu et al., 2011). Therefore, optimizing DO technology by shortening consolidation periods and improving new bone quality (mineralization, microstructure, and mechanical strength) is of significant clinical importance.

During DO, mechanical distraction triggers a complex cascade of biological responses, including localized inflammation, hypoxia, and growth factor release (Yang et al., 2022). This microenvironmental milieu activates multiple signaling pathways, including SDF-1/CXCR4, PDGF, and TGF-β signaling, which collectively promote bone marrow mesenchymal stem cell (BMSC) recruitment from both local bone marrow and systemic circulation (Liu et al., 2013). The inflammatory response, characterized by elevated IL-1β, TNF-α, and IL-6 levels, creates chemotactic gradients, facilitating MSC homing while influencing differentiation potential through the modulation of key transcription factors (Yang et al., 2022). BMSCs serve as key seed cells for bone tissue regeneration, possessing multipotent differentiation capacity and secreting cytokines involved in bone formation, angiogenesis, and immune modulation (Polymeri et al., 2016; Zhang et al., 2022; Zhang et al., 2021; Wu et al., 2025). Therefore, carefully modulating BMSC biological behaviors in a controlled manner, while maintaining cellular homeostasis to prevent unwanted effects such as ectopic bone formation, represents a promising approach to enhance DO efficiency.

Calcitonin gene-related peptide (CGRP), a 37-amino acid neuropeptide widely distributed in sensory nerve terminals, primarily exerts biological effects through binding to its receptor complex composed of CALCRL and RAMP1 (Russell et al., 2014; Liang et al., 2018). Beyond its traditional vasodilatory roles, CGRP plays complex and context-dependent roles in bone metabolism. Although CGRP promotes osteoblast proliferation and differentiation and inhibits osteoclast formation in mature bone tissue (Kee et al., 2018; Leroux et al., 2024), its effects during bone repair processes may differ. Initial bone resorption can facilitate BMSC recruitment and repair initiation, yet CGRP’s antiresorptive effects could theoretically influence early regeneration phases. However, recent evidence suggests that CGRP’s bone anabolic effects predominate during active repair, with studies demonstrating enhanced fracture healing and bone regeneration following CGRP treatment (He et al., 2016; Wang et al., 2010; Xu et al., 2020). In DO models, preliminary studies indicate CGRP participation in new bone formation regulation although specific effects on BMSC biology and underlying molecular mechanisms remain incompletely understood (Mi et al., 2021).

Cyclic adenosine monophosphate (cAMP), as an important intracellular second messenger, participates in regulating various cellular functions. The cAMP-dependent protein kinase A (PKA) signaling pathway represents a classical intracellular signal transduction pathway (Vitali et al., 2014). Upon activation, PKA phosphorylates multiple downstream substrates, including cAMP response element-binding protein (CREB). Phosphorylated CREB (p-CREB), as an important nuclear transcription factor, can bind to cAMP response elements (CREs) in target gene promoter regions, regulating transcriptional expression of these genes and subsequently affecting multiple biological processes, including cell proliferation, differentiation, apoptosis, and survival (Kida, 2012; Molnar et al., 2014). Previous studies have demonstrated that the cAMP/PKA/CREB signaling pathway plays crucial roles in BMSC osteogenic differentiation (Jiang et al., 2025; Chen et al., 2016). Some studies suggest that CGRP may exert biological effects through activating the cAMP/PKA signaling pathway (Holzmann, 2013; García-Morales et al., 2017). However, whether CGRP regulates BMSC osteogenic differentiation through the cAMP/PKA/CREB signaling pathway and subsequently affects bone regeneration during DO currently lacks systematic investigation.

Based on this background, we hypothesized that CGRP enhances BMSC proliferation, migration, antiapoptotic capacity, and osteogenic differentiation through cAMP/PKA/CREB pathway activation, thereby improving DO efficiency and new bone quality. In this study, we represent the first systematic investigation of CGRP’s mechanisms in DO, a specialized bone regeneration modality that creates unique biomechanical and biochemical microenvironments distinct from standard fracture healing. Our research investigated CGRP effects on rat BMSC osteogenic differentiation *in vitro* and bone regeneration promotion in SD rat femoral DO models *in vivo*, with emphasis on elucidating cAMP/PKA/CREB signaling pathway involvement.

## 2 Materials and methods

### 2.1 Ethical statement

All experimental protocols were approved by the Institutional Animal Care and Use Committee of Xinjiang Medical University (Approval Number: IACUC-JT-20231121-35) and conducted in accordance with the Guidelines for the Care and Use of Laboratory Animals.

### 2.2 Materials and reagents

Rat α-calcitonin gene-related peptide (rat α-CGRP, HY-P0203), CGRP receptor antagonist rat CGRP8-37 (HY-P0209), and PKA inhibitor H89 (HY-15979) were purchased from MCE (China). CGRP8-37 is a well-characterized competitive antagonist of CGRP receptors that blocks CGRP binding to its receptor complex (CALCRL/RAMP1) without intrinsic agonist activity. H89 is a potent and selective inhibitor of PKA that competitively inhibits ATP binding to the catalytic subunit, thereby blocking cAMP-dependent PKA activation and downstream CREB phosphorylation.

### 2.3 *In vitro* cell studies

#### 2.3.1 Cell isolation and culture

Six-week-old healthy male Sprague–Dawley (SD) rats (n = 8) were euthanized by intraperitoneal injection of excess pentobarbital sodium (150 mg/kg). Under aseptic conditions, femurs and tibias were rapidly isolated with careful removal of attached muscles and connective tissues. Bones were washed in PBS containing 1% penicillin–streptomycin (100 units/mL penicillin and 100 μg/mL streptomycin). In a laminar flow hood, bone epiphyses were cut, and bone marrow cavities were repeatedly flushed with α-MEM medium containing 10% FBS (Gibco, Invitrogen, US) and 1% penicillin–streptomycin (100 units/mL penicillin and 100 μg/mL streptomycin; Gibco, Invitrogen, US) to collect bone marrow cell suspension. Cell suspensions were gently pipetted several times through 22-gauge needles to disperse cell clumps and filtered through 100 μm cell strainers to remove bone fragments and tissue debris. Filtered cell suspensions were seeded in T25 culture flasks at 1 × 10^6^ to 1 × 10^7^ cells/mL density and cultured in a humidified incubator (Thermo Fisher, 3111) at 37 °C with 5% CO_2_. First medium change occurred 24–48 h post-seeding to remove non-adherent cells, followed by medium changes every 2–3 days. When cells reached 80%–90% confluence, they were digested with 0.25% trypsin–EDTA and subcultured at a ratio of 1:2. Fourth-passage BMSCs were used for all subsequent *in vitro* experiments to ensure cell source homogeneity and experimental result stability.

#### 2.3.2 BMSC characterization

Fourth-passage BMSCs in the logarithmic growth phase were digested with 0.25% trypsin–EDTA to prepare single-cell suspensions. Cell concentration was adjusted to 1 × 10^6^ cells/mL. A 500-μL cell suspension was incubated with 5 μL each of APC-CD29 (BioLegend, United States), PE-CD105 (BioLegend, United States), PE-CD45 (BioLegend, United States), and PE-CD90 monoclonal antibodies (BioLegend, United States), along with isotype controls. After 30 min of incubation at room temperature in the dark, the cells were washed with PBS and centrifuged at 1,000 rpm for 5 min, and the supernatant was discarded. The cells were resuspended in 500 μL PBS and analyzed using flow cytometry (BD Biosciences, United States), with FlowJo software used to analyze CD29, CD105, CD45, and CD90 expressions.

#### 2.3.3 Multipotent differentiation assessment

Fourth-passage BMSCs were seeded at 2 × 10^4^ cells/well in six-well plates. Upon reaching 60%–70% confluence, cells were switched to osteogenic, adipogenic, and chondrogenic induction media (OriCell, Cyagen Biosciences, Guangzhou, China). BMSCs cultured in basic medium (α-MEM without induction components) served as controls. After 21 days of osteogenic induction, Alizarin Red S staining was performed to observe calcium nodule formation. After 14 days of adipogenic induction, Oil Red O staining was performed to observe lipid droplet formation. After 21 days of chondrogenic induction, Alcian Blue staining was performed to observe proteoglycan accumulation. All staining results were observed and photographed under an inverted microscope (Leica DMi8, Berlin, Germany).

#### 2.3.4 Cell proliferation assay

CCK-8 assay was used to detect CGRP effects on BMSC proliferation. Fourth-passage BMSCs were seeded at 1 × 10^4^ cells/well in 96-well plates with 100 µL per well. After 24 h of cell attachment, the old medium was discarded, and fresh medium containing different CGRP concentrations (10^−7^, 10^−8^, 10^−9^, 10^−10^, and 10^−11^ M) was added, along with blank medium controls. Plates were placed in 37 °C, 5% CO_2_ incubators for continued culture. On days 1, 3, 5, and 7, the medium was removed, and 100 μL of complete medium containing 10% CCK-8 was added to each well, followed by 1–2 h of incubation. Absorbance at 450 nm was measured using a multifunctional microplate reader (Beijing Pulang New Technology Co., Ltd., DNM-9602). Each experiment included three technical replicates per treatment group, and all experiments were independently repeated three times (n = 3 independent experiments). Based on the results, the optimal CGRP concentration for subsequent experiments was determined.

#### 2.3.5 Apoptosis analysis

An Annexin V-FITC Apoptosis Detection Kit (Beyotime Biotechnology, China) was used to observe BMSC apoptosis after different treatments. Fourth-passage BMSCs were seeded at 1 × 10^5^/well in six-well plates. After cell attachment, four groups were established: control (blank medium), CGRP (10^−8^ M), CGRP + CGRP8-37 (10^−8^ M CGRP + 10^−6^ M CGRP8-37), and CGRP + H89 (10^−8^ M CGRP + 0.2 μg/mL H89). For combined treatments, cells were pre-incubated with CGRP8-37 or H89 for 30 min prior to CGRP addition. Fresh inhibitors were replenished with each medium change throughout the experimental period to maintain consistent inhibitory effects. After 6 h of culture, serum-free medium was used to induce apoptosis, with cell collection at 12 and 24 h. Collected cells were washed twice with precooled PBS and resuspended in 500 μL binding buffer. A volume of 5 μL of Annexin V-FITC and 5 μL of PI were added, gently mixed, and incubated at room temperature in the dark for 10–15 min. Flow cytometry was immediately used to detect apoptosis, analyzing early apoptotic (Annexin V^+^/PI^−^) and late apoptotic (Annexin V^+^/PI^+^) cell percentages. The total apoptotic rate was calculated as the sum of early apoptotic and late apoptotic cell percentages (Annexin V^+^/PI^−^ + Annexin V^+^/PI^+^), representing all cells undergoing apoptotic cell death, regardless of stage.

#### 2.3.6 Live/dead cell fluorescent staining

A Calcein AM/PI Fluorescent Staining Kit [Engineering for Life (EFL), Suzhou, China] was used to observe BMSC growth activity after different treatments. Calcein AM detects intracellular esterase activity, whereas PI detects cell membrane integrity for biocompatibility assessment. Cells were seeded at 2 × 10^4^ cells/well in 12-well plates. After cell attachment, grouping was performed as described in section 2.3.5. After 1 and 3 days of culture, cells were carefully washed three times with PBS, fixed with 4% paraformaldehyde (PFA) for 20 min at room temperature, and washed again with PBS. Following the Calcein AM/PI Live/Dead Cell Double Staining Kit instructions, 500 μL working solution containing 0.5% Calcein AM was added to each well for 10 min at 37 °C in the dark; after PBS washing, 200 μL working solution containing 0.25% PI was added for 5 min at room temperature in the dark, followed by PBS washing. Images were captured using an inverted fluorescence microscope (Leica, Berlin, Germany), with live cells showing green fluorescence (Calcein AM-positive) and dead cell nuclei showing red fluorescence (PI-positive). Each experiment included three technical replicates per treatment group, and all experiments were independently repeated three times (n = 3 independent experiments).

#### 2.3.7 Cell morphology fluorescent staining

Phalloidin/DAPI staining was used to study BMSC morphology under different treatments. Phalloidin specifically binds cellular actin to label cell contours, whereas DAPI binds double-stranded DNA in cell nuclei. Cells were seeded at 2 × 10^4^ cells/well in 12-well plates. After cell attachment, grouping was performed as described in section 2.3.5. After 1 and 3 days of culture, cells were washed three times with PBS, fixed with 4% PFA for 15–20 min at room temperature, washed with PBS (three times, 5 min each), permeabilized with 0.5% Triton X-100 for 10 min at room temperature, washed with PBS, and blocked with 1% BSA for 30 min at room temperature. FITC-labeled phalloidin (5 μg/mL) working solution was then added and incubated for 30 min at room temperature in the dark to label F-actin. After PBS washing (three times, 5 min each), DAPI (100 ng/mL) mounting medium was used for nuclear counterstaining and mounting. Cell morphology, spreading, and F-actin distribution were observed using an inverted fluorescence microscope (Leica DMi8) with image recording. Each experiment included three technical replicates per treatment group, and all experiments were independently repeated three times (n = 3 independent experiments).

#### 2.3.8 Transwell migration assay

Transwell chambers with 8.0 μm pore-sized polycarbonate membrane inserts (Corning, United States) were used to evaluate the effects of different treatments on BMSC migration ability. BMSCs in the logarithmic growth phase were digested and resuspended in serum-free α-MEM medium at 5 × 10^5^ cells/mL density. A 200-μL cell suspension (containing 1 × 10^5^ cells) was seeded in Transwell upper chambers. Lower chambers contained 600 μL medium with different treatments (grouping was performed as described in section 2.3.5) containing 10% FBS as an chemoattractant. Culture plates were placed in 37 °C, 5% CO_2_ incubators for 24 h. After culture, upper chambers were removed, and non-migrated cells on the inner surfaces of the upper chamber were gently wiped with moist cotton swabs. Cells that migrated to the membrane outer sides of the lower chamber were fixed with methanol for 30 min at room temperature and then stained with 0.1% crystal violet solution for 15–20 min. After washing excess dye with PBS (three times, 2 min each), images were captured under an inverted microscope, and migrated cells were counted. Each experiment included three technical replicates per treatment group, and all experiments were independently repeated three times (n = 3 independent experiments).

#### 2.3.9 Scratch assay

Scratch assays evaluated effects of different treatments on BMSC lateral migration ability. BMSCs in the logarithmic growth phase were collected and seeded at 2 × 10^5^ cells/well in 24-well plates and then cultured until approximately 90%–100% confluence to form monolayers. Sterile 200-μL pipette tips were used to rapidly scratch across cell monolayer centers, creating uniform-width scratches. Cells were gently washed 2–3 times with PBS to remove detached cells and debris. Medium containing different treatments (grouping was performed as described in section 2.3.5) was then added to each well. Scratch area images were captured at the same positions at 0 h (immediate), 12 h, and 24 h using an inverted microscope. ImageJ software (National Institutes of Health, Bethesda, MD, United States) was used to measure and calculate the scratch area, with cell migration ability assessed by comparing scratch closure degrees at different time points. Each experiment included three technical replicates per treatment group, and all experiments were independently repeated three times (n = 3 independent experiments).

#### 2.3.10 Alizarin red staining

Alizarin Red S (ARS) staining was used to assess extracellular matrix mineralization after BMSC osteogenic differentiation under different treatment conditions. BMSCs were seeded at 2 × 10^4^ cells/well in 12-well plates. After cell attachment, osteogenic induction medium (OriCell, Cyagen Biosciences) containing different treatments (grouping was performed as described in section 2.3.5) was added. After 14 and 21 days of culture, the medium was discarded, and cells were washed three times with PBS. Cells were fixed with 4% paraformaldehyde for 15–20 min at room temperature and then washed once with PBS. A volume of 1 mL of 1% (w/v) ARS solution (Beyotime Biotechnology, China, pH 4.1–4.3) was added per well for 20–30 min of staining at room temperature. After staining, excess dye was carefully rinsed with distilled water (3 times, 2 min each) until a clear background was achieved. Orange or deep red calcified nodule formation was observed and photographed under an inverted microscope. Each experiment included three technical replicates per treatment group, and all experiments were independently repeated three times (n = 3 independent experiments). For quantitative analysis of mineralization, bound Alizarin Red dye was extracted using 10% cetylpyridinium chloride (CPC) solution for 1 h at room temperature with gentle agitation. The extracted solution was transferred to 96-well plates, and absorbance was measured at 562 nm using a microplate reader. ARS content was calculated and normalized per well.

#### 2.3.11 Alkaline phosphatase (ALP) staining

ALP is an important early marker of osteoblast differentiation. BMSCs were seeded at 2 × 10^4^ cells/well in 12-well plates. After cell attachment, osteogenic induction medium containing different treatments (grouping was performed as described in section 2.3.5) was added. ALP staining was performed after 4 and 7 days of culture. Medium was discarded, and cells were washed three times with PBS, fixed with 4% paraformaldehyde for 15 min at room temperature, and then washed once with PBS. BCIP/NBT Alkaline Phosphatase Color Development Kit (Beyotime Biotechnology, China) instructions were followed: an appropriate amount of BCIP/NBT working solution was added to each well and incubated at room temperature in the dark for 15–30 min until an obvious purple-blue precipitate appeared. After reaction termination, excess dye solution was rinsed with PBS (three times, 2 min each) to remove the unbound substrate. Purple deposits in cells or cell colonies were observed and photographed under an inverted microscope. Each experiment included three technical replicates per treatment group, and all experiments were independently repeated three times (n = 3 independent experiments). For quantitative ALP activity measurement, cells were lysed with RIPA buffer containing protease inhibitors. Cell lysates were incubated with p-nitrophenyl phosphate (p-NPP) substrate solution at 37 °C for 30 min. The reaction was terminated with 2M NaOH, and absorbance was measured at 405 nm. ALP activity was calculated as units per milligram of total protein, with the total protein content determined using the BCA assay.

#### 2.3.12 cAMP ELISA detection

To detect CGRP effects on intracellular second messenger cAMP levels, a cAMP ELISA Kit (Elabscience, Wuhan, China) was used. BMSCs were seeded at 2 × 10^5^ cells/well in six-well plates. After cell attachment, grouping was performed as described in section 2.3.5 (control, CGRP, CGRP + CGRP8-37, and CGRP + H89 groups). After 30 min of treatment, cells from each group were collected. The cells were first washed twice with precooled PBS, and then, cell lysis buffer was added for 10–15 min of lysis on ice. Cell lysate was collected and centrifuged to remove the cell debris. The supernatant was collected and detected according to cAMP ELISA kit instructions. In brief, samples or standards were added to pre-coated antibody plates, incubated, and washed; enzyme conjugate was then added, followed by another incubation and wash; finally, substrate (Ellman reagent) was added for color development. Absorbance values were measured using a microplate reader. cAMP concentrations in samples were calculated according to the standard curves and corrected by total protein concentration measured using the BCA method. Three independent experiments were performed.

#### 2.3.13 Quantitative real-time PCR (qRT-PCR)

qRT-PCR was used to analyze mRNA expression levels of osteogenesis- and pathway-related genes. BMSCs were seeded at 2 × 10^5^ cells/well in six-well culture plates. After cell attachment, osteogenic induction medium containing different treatments (grouping was performed as described in section 2.3.5) was added. After 7 days of culture, cells from each group were collected, and the total RNA was extracted using TRIzol reagent. RNA concentration and purity were measured using a NanoDrop spectrophotometer (A260/A280 ratio between 1.8 and 2.0). One microgram of total RNA was reverse-transcribed into cDNA using PrimeScript™ RT Master Mix. Diluted cDNA was used as a template for amplification using SYBR^®^ Premix Ex Taq™ II (TaKaRa, Japan) on a real-time fluorescent quantitative PCR instrument (Thermo Fisher Scientific, FHG-PLUS). Target genes included key osteogenic differentiation transcription factors *Runx2* (Runt-related transcription factor 2) and *Sp7* (Osterix, Osx), late osteogenic marker genes *Spp1* (secreted phosphoprotein 1, osteopontin gene, OPN) and *Bglap* (bone gamma-carboxyglutamate protein, osteocalcin gene, OCN), and cell cycle regulatory gene *CyclinD1*. *Gapdh* (glyceraldehyde-3-phosphate dehydrogenase) served as an internal reference gene for normalization. Primer sequences were designed using Primer Premier 5.0 software and are listed in Table 1. All primers were synthesized by Sangon Biotech (Shanghai, China) and validated for specificity and efficiency. Each sample had three technical replicates. The relative expression of target genes was calculated using the 2^−ΔΔCt^ method.

**TABLE 1:** Primer sequences used for quantitative real-time PCR analysis.

Gene	Forward primer (5′-3′)	Reverse primer (5′-3′)	Product size (bp)
*Runx2*	TCG​TCA​GCG​TCC​TAT​CAG​TTC​C	CTT​CCA​TCA​GCG​TCA​ACA​CCA​TC	110
*Sp7 (Osx)*	CTG​GCA​CAC​TGG​CGA​GAG​G	GCA​GAG​CAG​ACA​GGT​GAA​CTT​C	120
*Spp1 (OPN)*	CGA​TGA​TGA​CGA​CGA​CGA​TGA​C	CTT​GTG​TGC​TGG​CAG​TGA​AGG	95
*Bglap (OCN)*	GAC​CCT​CTC​TCT​GCT​CAC​TCT​G	CAC​CAC​CTT​ACT​GCC​CTC​CTG	103
*Ccnd1 (CyclinD1)*	CGC​CCT​CCG​TTT​CTT​ACT​TCA​AG	CCT​CGC​AGA​CCT​CTA​GCA​TCC	118
*Gapdh*	AAG​TTC​AAC​GGC​ACA​GTC​AAG​G	GAC​ATA​CTC​AGC​ACC​AGC​ATC​AC	143

#### 2.3.14 Western blot analysis

Western blot analysis was used to detect the phosphorylation levels of key proteins in the PKA/CREB signaling pathway and the expression of osteogenesis-related proteins and CyclinD1. For phosphorylated protein detection, BMSCs were cultured in regular medium to appropriate confluence, serum-starved for 12–24 h, and then grouped according to section 2.3.5 and stimulated with corresponding drugs for 30 min before cell collection. For total protein detection, BMSCs were cultured in the osteogenic induction medium for 7 days, as described in section 2.3.13; then, cells from each group were collected. RIPA lysis buffer containing protease and phosphatase inhibitors was added for 30 min of cell lysis on ice. Samples were centrifuged at 4 °C and 12,000 rpm for 15 min, and the supernatant was collected. Protein concentration was measured using the BCA method. Equal amounts of total protein (30 μg per lane) underwent SDS-PAGE gel electrophoresis using 10% or 12% polyacrylamide gels, and then, proteins were wet-transferred to PVDF membranes. Membranes were blocked with TBST buffer (tris-buffered saline with 0.1% Tween-20) containing 5% non-fat dry milk for 2 h at room temperature for most antibodies or 5% BSA in TBST for phospho-specific antibodies (p-PKA and p-CREB) for 2 h at room temperature to minimize background signals. Subsequently, PVDF membranes were incubated overnight at 4 °C with specific primary antibodies at the following dilutions: PKA antibody (1:1,000; Affinity, AF7746), phosphorylated PKA antibody (1:1,000; Affinity, AF7246), CREB antibody (1:1,000; Affinity, AF6188), phosphorylated CREB antibody (1:1,000; Affinity, AF3189), OPN antibody (1:1,000; Affinity, AF0227), OCN antibody (1:1,000; Affinity, DF12303), Runx2 antibody (1:1,000; Abcam, ab236639, Cambridge, United Kingdom), Osx antibody (1:1,000; Abcam, ab209484), CyclinD1 antibody (1:1,000; Abcam, ab134175), and GAPDH antibody (1:2000; Abcam, ab181602). Next day, membranes were washed three times with TBST, 10 min each. Subsequently, the HRP-labeled goat anti-rabbit IgG secondary antibody (Abcam, ab6721) was incubated for 1 h at room temperature. After thorough TBST washing, an enhanced chemiluminescence (ECL) kit was used to detect antibody-bound protein bands on a chemiluminescence imaging system (Azure Biosystems C600, United States). ImageJ software (National Institutes of Health, United States) was used for quantitative analysis of protein band optical density values. Phosphorylated protein levels were expressed as ratios to corresponding total proteins, whereas other target protein expression levels were normalized to GAPDH ratios. Additional experimental details, including complete Western blot images and control experiments, are provided in the Supplementary Materials. Each experiment included three technical replicates per treatment group, and all experiments were independently repeated three times (n = 3 independent experiments).

### 2.4 *In vivo* experiments

#### 2.4.1 Experimental animals and grouping

Adult male SD rats weighing approximately 400–450 g were provided by the Xinjiang Medical University Experimental Animal Center. All rats were housed in SPF-grade animal facilities under standard environmental conditions: temperature 22 °C ± 2 °C, humidity 50% ± 10%, and 12-h light/12-h dark cycle. Animals underwent adaptive feeding for at least 7 days with free access to standard pellet feed and sterile drinking water. A total of 108 rats were randomly divided into four groups (n = 27/group): control group: local injection of 100 μL saline into the distraction gap every other day during the 10-day distraction period. CGRP group: local injection of 100 μL saline containing 10^−8^ M CGRP into the distraction gap every other day during the 10-day distraction period. CGRP + CGRP8-37 group: local injection of 100 μL saline containing 10^−8^ M CGRP and 10^−6^ M CGRP8-37 into the distraction gap. CGRP + H89 group: local injection of 100 μL saline containing 10^−8^ M CGRP and 10 μM H89 into the distraction gap.

#### 2.4.2 Rat femoral distraction osteogenesis model establishment and intervention

All surgical procedures were performed by the same experienced technical team to reduce the experimental error. Rats were fasted for 12 h pre-surgery without water restriction. Anesthesia was induced by an intraperitoneal injection of 2% pentobarbital sodium (3 mg/100 g body weight). The right hind limb femoral region was shaved and disinfected. Benzathine penicillin (200,000 units/animal) was injected intramuscularly 30 min pre-surgery for infection prevention. Under aseptic conditions, a longitudinal skin incision of approximately 2 cm long was made on the right femoral lateral side, and muscles were bluntly dissected to expose the mid-femoral shaft. A custom unilateral mini external fixator was securely fixed to proximal and distal femoral shafts using four 1.2-mm-diameter stainless steel self-tapping screws. Subsequently, precise transverse osteotomy was performed at the femoral shaft midpoint perpendicular to the long axis of the femur between two pairs of fixation pins using a mini oscillating saw or high-speed burr, with care to protect surrounding soft tissues and blood supply. After osteotomy completion, the surgical area was thoroughly irrigated with sterile saline to confirm complete osteotomy. Muscles, fascia, and skin were sutured layer by layer. Postoperative X-rays were immediately taken to confirm the osteotomy position and external fixator installation stability. Benzathine penicillin (200,000 units/animal/day) was injected intramuscularly for 3 consecutive postoperative days. Daily examination and cleaning of external fixation pin sites were performed, and antibiotic ointment was applied. Rats were housed individually with free movement, feeding, and drinking. After a 5-day latency period, femoral distraction began. The distraction rate was 0.25 mm/session, twice daily (12-h intervals), for a total distraction distance of 0.5 mm per day. Distraction continued for 10 days, ultimately creating a 5.0-mm distraction gap between femoral osteotomy ends. After completion of the distraction phase, a 6-week bone tissue mineralization consolidation period began.

Intervention measures: given the short half-life of CGRP (6–25 min due to peptidase degradation), repeated injections were necessary to maintain therapeutic effects. According to grouping, from postoperative day 6 to day 15 (corresponding to distraction days 1–10), every other day (a total of five injections per animal), and under isoflurane inhalation anesthesia and C-arm X-ray guidance, the corresponding 100 μL solutions were slowly and precisely injected into the central region of the distraction gap using microsyringes. The needle was maintained in position for 30 s after injection completion before slow withdrawal to allow tissue equilibration and reduce leakage. Additionally, the fibrous tissue naturally formed around the distraction gap during the latency period provides a semi-contained environment that helps retain injected solutions within the target area. The small injection volume (100 μL) and the controlled injection technique minimize the risk of significant leakage while ensuring adequate drug distribution throughout the distraction zone.

Sample collection: at 2, 4, and 6 weeks after the completion of the consolidation period, rats were euthanized by intraperitoneal injection of excess pentobarbital sodium. Right femurs were rapidly dissected with careful removal of surrounding soft tissues for subsequent detection and analysis. A total of 108 rats were randomly divided into four groups (n = 27/group), with animals allocated across different time points and experimental endpoints as follows: at 2 weeks post-consolidation, six animals per group were allocated for histological analysis (three for undecalcified sections and three for decalcified sections and immunohistochemistry). At 4 weeks post-consolidation, six animals per group were similarly allocated for histological analysis. At 6 weeks post-consolidation, the remaining 15 animals per group were distributed as follows: three animals were used sequentially for gross morphology documentation, radiological examination, micro-CT analysis, and subsequently biomechanical testing (allowing comprehensive assessment of the same specimens); six animals for histological assessment (three for undecalcified sections and three for decalcified sections and immunohistochemistry); and six animals for additional validation studies and potential backup analyses. This distribution ensured adequate sample sizes for each analytical endpoint while accounting for potential experimental variability and maintaining statistical power for group comparisons.

#### 2.4.3 Radiological examination

All experimental rats, under brief isoflurane anesthesia, received regular anteroposterior (AP) X-ray examinations of distraction areas from immediately post-surgery (typically immediately postoperative, then weekly) until euthanasia. All X-ray images were taken using the same digital X-ray equipment (HF400VA, MIKASA X-RAY Co., Ltd., Tokyo, Japan) under strictly controlled identical conditions (exposure parameters: 44 kV and 4.5 mAs) to ensure image quality consistency and result comparability for dynamic monitoring of new bone callus formation, density changes, and overall bone healing progress within distraction gaps.

#### 2.4.4 Micro-CT scanning and analysis

After 6 weeks of consolidation period completion, three right femoral specimens from each experimental group were selected and fixed in 4% paraformaldehyde solution for 48 h. Subsequently, a high-resolution micro-computed tomography (micro-CT) scanner (SkyScan 1176, Bruker Micro-CT, Rheinstetten, Germany) was used for three-dimensional imaging and quantitative assessment of new bone in distraction areas. Scanning parameters: Al + Cu filter, voxel size 18 μm, 65 kV, 385 μA, scanning time 340 ms. Raw data obtained from scanning underwent image reconstruction using accompanying Skyscan NRecon software. Subsequent three-dimensional analysis used Skyscan CTAn software, selecting the entire distraction region formed between femoral osteotomy ends as region of interest (ROI). Within ROI, a series of key bone microstructural parameters were precisely measured and calculated, including bone mineral density (BMD, g/cm^3^), bone volume fraction (BV/TV, %), trabecular number (Tb.N, mm^−1^), trabecular thickness (Tb.Th, μm), and trabecular separation (Tb.Sp, μm) for comprehensive evaluation of new bone quality and structural characteristics.

#### 2.4.5 Biomechanical testing

Within 24 h after animal euthanasia, at 6 weeks post-consolidation, right femoral specimens from three rats per group (after external fixator removal and careful surrounding soft tissue dissection) underwent biomechanical performance testing to assess new bone mechanical strength. Contralateral unoperated left femurs served as internal controls. Femoral specimens were carefully placed on three-point bending testing machine platforms (JD-D500/JD-T100, China National Machinery Testing Equipment, China) with support point span set at 18 mm. Loading head was precisely aligned with the distraction area midpoint, applying loads in the AP direction at a constant loading rate of 0.5 mm/min until obvious specimen fracture. Load–displacement curves were recorded in real-time settings throughout loading. Key mechanical parameters were calculated and analyzed based on the following curve data: ultimate load (N), energy to failure (mJ), and stiffness (N/mm). Data could be compared and normalized with corresponding parameters from contralateral healthy femurs.

#### 2.4.6 Histomorphological and immunohistochemical analyses

At predetermined time points (2, 4, and 6 weeks post-consolidation), right femoral specimens were collected for histological and immunohistochemical (IHC) analyses. Undecalcified hard tissue sections: at each time point, three femoral specimens were randomly selected from each group, fixed in 10% neutral formalin for 48 h, and then transferred to 75% ethanol for storage. Specimens underwent gradient alcohol dehydration and xylene clearing, followed by methyl methacrylate (MMA) embedding. Using hard tissue sectioning machine (HistoCore AUTOCUT, Leica, Wetzlar, Germany), 10-μm-thick longitudinal undecalcified bone tissue sections were prepared. These sections underwent Von Kossa staining (observing mineralized bone tissue distribution and extent), Masson trichrome staining (distinguishing collagen fibers and bone tissue), Goldner trichrome staining (distinguishing mineralized bone, osteoid matrix, and cellular components), and safranin O–fast green staining (observing cartilage tissue presence and distribution). After staining completion, new bone morphological characteristics, quantity, maturity, and connections with host bone were observed under optical microscopy. Decalcified bone tissue sections and IHC staining: at each time point, three additional femoral specimens from each group were fixed and then decalcified in 10% EDTA solution (pH 7.4) for approximately 4 weeks (fresh decalcification solution changed weekly) until complete bone tissue softening. Completely decalcified specimens underwent conventional gradient alcohol dehydration, xylene clearing, and paraffin embedding; then, they were cut into 5-μm-thick consecutive longitudinal sections using a rotary microtome (RM2135, Leica, Wetzlar, Germany). After deparaffinization and rehydration, antigen retrieval was performed in citrate buffer (10 mM sodium citrate, pH 6.0) using a microwave oven at 95 °C for 15 min, followed by natural cooling to room temperature for 30 min. Endogenous peroxidase was inactivated by incubating the sections in 3% H_2_O_2_ solution for 10–15 min at room temperature. Blocking was performed with 5% BSA or normal goat serum for 30 min at room temperature. Primary antibody working solutions were then applied, including rabbit polyclonal anti-Runx2 (1:200; Bioss, bs-1134R, Woburn, MA, United States), rabbit polyclonal anti-Osterix (Osx) (1:100; ServiceBio, GB111900, Wuhan, China), mouse monoclonal anti-osteocalcin (OCN) (1:150; ServiceBio, GB115684), rabbit polyclonal anti-osteopontin (OPN) (1:200; ServiceBio, GB11500), rabbit monoclonal anti-phosphorylated PKA (p-PKA Thr197) (1:100; Affinity Biosciences, #AF7246), and rabbit monoclonal anti-phosphorylated CREB (p-CREB Ser133) (1:100; Affinity Biosciences, #AF3189, Cincinnati, OH, United States), with sections placed in humid chambers at 4 °C overnight. Next day, after thorough PBS washing, appropriate HRP-labeled secondary antibodies were applied: goat anti-rabbit IgG (1:5,000; ServiceBio, GB23303) for rabbit primary antibodies or goat anti-mouse IgG (1:5,000; ServiceBio, GB23301) for mouse primary antibodies, and incubated for 1 h at room temperature. After PBS washing again, a DAB Color Development Kit was used for visualization. Hematoxylin counterstained nuclei. Finally, sections underwent dehydration and clearing; then, they were mounted with neutral balsam. Target protein positive staining in sections from each group was observed under an optical microscope (×200 magnification). For quantitative analysis of immunohistochemical staining, five random high-power fields (×200 magnification) per section were photographed using standardized exposure settings. Positive staining intensity was quantified using ImageJ software (National Institutes of Health, United States), with the following protocol: images were converted to RGB color images, and brown DAB-positive staining was isolated using color threshold analysis. The integrated optical density (IOD) of positive staining was measured and normalized to the total area of each field. The mean IOD/area ratio was calculated for each animal, with three sections per animal and five animals per group analyzed for statistical evaluation.

### 2.5 Statistical analysis

All experimental data were analyzed using the SPSS 22.0 statistical software package (SPSS Inc., Chicago, IL, United States). Quantitative data were expressed as mean ± standard deviation (x ± s). The Shapiro–Wilk test first assessed the normal distribution of each group’s data. For normally distributed data with equal variances, an independent samples t-test was used for two-group comparisons; one-way analysis of variance (one-way ANOVA) was used for multi-group comparisons, with Tukey’s HSD or LSD *post hoc* tests for pairwise comparisons if ANOVA showed statistical significance. For non-normally distributed data or unequal variances, the Mann–Whitney U test was used for two independent sample comparisons; the Kruskal–Wallis H test was used for multi-group comparisons, with Bonferroni-corrected Mann–Whitney U tests for *post hoc* pairwise comparisons if statistically significant. For immunohistochemical quantification, integrated optical density data were analyzed using one-way ANOVA, followed by Tukey’s HSD *post hoc* test for multiple comparisons between groups. Data normality was assessed using the Shapiro–Wilk test prior to analysis. All statistical tests were two-sided, with P < 0.05 considered statistically significant. Experimental figures and charts were created using GraphPad Prism v.8.0 software (GraphPad Software Inc., San Diego, CA, United States).

## 3 Results

### 3.1 CGRP effects on *in vitro* BMSC functions

#### 3.1.1 BMSC culture and characterization

**FIGURE 1:**
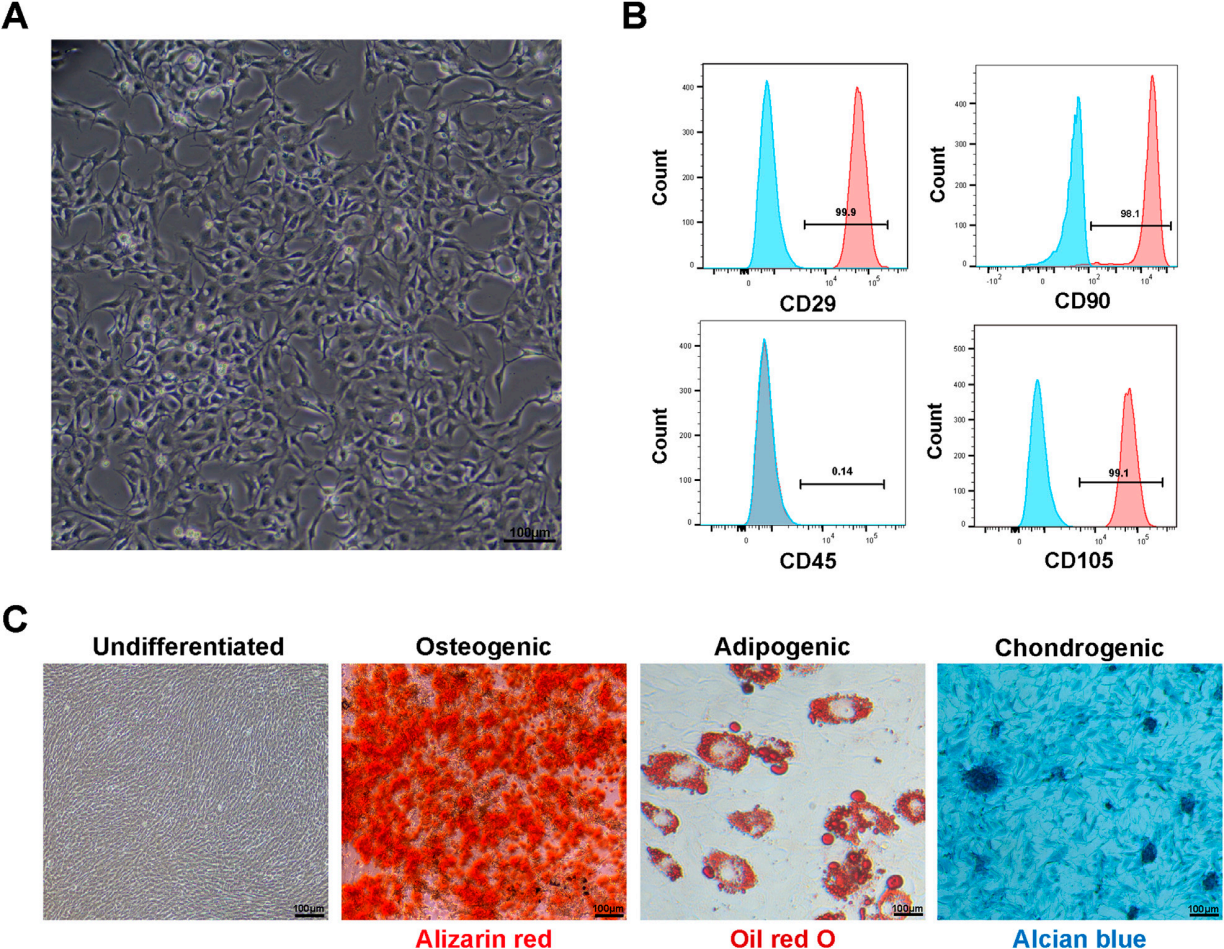
Characterization of rat bone marrow mesenchymal stem cells (BMSCs): **(A)** microscopy showing typical spindle-shaped morphology of fourth-passage BMSCs cultured for 48 h (scale bar: 100 μm). **(B)** Flow cytometric analysis demonstrating expression of mesenchymal stem cell surface markers. Fourth-passage BMSCs showed high expression of CD29 (95.9%), CD90 (98.1%), and CD105 (99.1%) with minimal expression of hematopoietic marker CD45 (0.14%), confirming mesenchymal stem cell identity according to ISCT criteria. **(C)** Multipotent differentiation capacity of BMSCs. Undifferentiated: BMSCs cultured in basic medium without induction factors showing typical fibroblast-like morphology with no spontaneous differentiation. Osteogenic: Alizarin Red S staining after 21 days of osteogenic induction showing abundant orange-red calcified nodule formation. Adipogenic: Oil Red O staining after 14 days of adipogenic induction showing numerous red lipid droplets within cells. Chondrogenic: Alcian Blue staining after 21 days of chondrogenic induction showing blue proteoglycan deposits in extracellular matrix (scale bars: 100 μm).

Primary cultured BMSCs began adhering 24–48 h post-seeding, exhibiting typical elongated spindle, fusiform, or irregular triangular morphology with clear nuclei and abundant cytoplasm, growing in colony-like or whirlpool arrangements (Figure 1A). Flow cytometry results showed that fourth-passage BMSCs highly expressed mesenchymal stem cell surface markers CD29 (average positive rate >95%), CD105 (average positive rate >95%), and CD90 (average positive rate >95%), while showing minimal expression of the hematopoietic stem cell surface marker CD45 (average positive rate <5%) (Figure 1B), consistent with BMSC phenotypic characteristics. Multipotent differentiation experiments (Figure 1C) showed that after 21 days of osteogenic induction, Alizarin Red S staining revealed abundant orange-red calcified nodule formation; after 14 days of adipogenic induction, Oil Red O staining showed numerous red lipid droplets within cells; after 21 days of chondrogenic induction, Alcian Blue staining revealed blue proteoglycan deposits in the extracellular matrix. The control group cells showed no obvious differentiation signs. These results demonstrated that successfully isolated and cultured cells possessed typical BMSC characteristics, providing solid foundation for subsequent experiments.

#### 3.1.2 CGRP effects on BMSC proliferation

**FIGURE 2:**
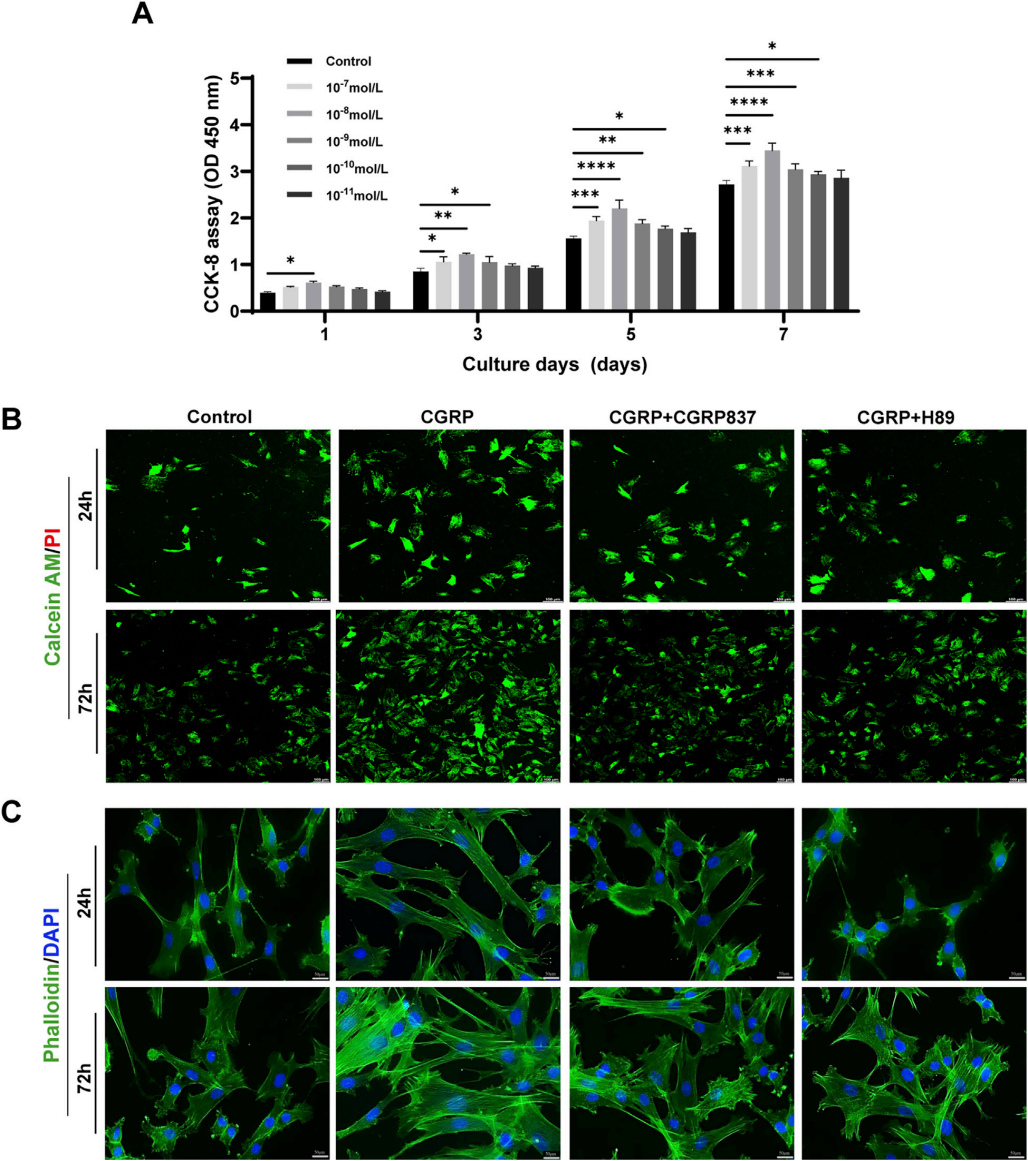
CGRP enhances BMSC proliferation and cellular morphology: **(A)** CCK-8 proliferation assay showing dose- and time-dependent effects of CGRP (10^−11^ to 10^−7^ M) on BMSC growth over 7 days. Data represent mean ± SD. *P < 0.05. **(B)** Calcein AM/PI live/dead staining at days 1 and 3, demonstrating enhanced cell viability with CGRP treatment. Green: live cells; red: dead cells (scale bars: 100 μm). **(C)** Phalloidin/DAPI staining revealing improved cell spreading and F-actin organization. Green: F-actin; blue: nuclei (scale bars: 50 μm).

CCK-8 assay results showed that different CGRP concentrations (10^−11^ M to 10^−7^ M) promoted BMSC proliferation to varying degrees in time- and dose-dependent manners (Figure 2A). On days 3, 5, and 7 of culture, compared with controls, 10^−9^ M, 10^−8^ M, and 10^−7^ M CGRP treatment groups showed significantly increased cell OD values (P < 0.05). Among these, 10^−8^ M CGRP demonstrated strongest proliferation-promoting effects at all time points, particularly on days 5 and 7. Therefore, 10^−8^ M was selected as optimal CGRP concentration for subsequent experiments. This finding suggests that CGRP can effectively stimulate BMSC proliferation, potentially providing more abundant seed cells for bone repair.

#### 3.1.3 CGRP effects on BMSC survival and morphology

Calcein AM/PI live/dead cell staining results (Figure 2B) showed that after 1 and 3 days of culture, all treatment groups displayed predominantly green fluorescence (Calcein AM-positive, live cells) with minimal red fluorescence signals (PI-positive, dead cell nuclei), indicating overall good cell viability across all experimental conditions. Although CGRP-treated cells maintained excellent viability, careful examination revealed slightly increased PI-positive signals in CGRP + CGRP8-37 and CGRP + H89 groups compared to CGRP alone, with the overall cell survival remaining high in all groups.

Phalloidin/DAPI staining observing cell morphology and F-actin distribution (Figure 2C) showed that after 1 and 3 days of culture, CGRP-treated BMSCs exhibited more extensive spreading with more elongated cell morphology and clear filamentous pseudopodia. Intracellular F-actin microfilament bundles were thicker with more ordered arrangements, forming good intracellular tension structures. Control group cells showed relatively lower spreading degrees and F-actin organization. CGRP co-treatment with CGRP8-37 or H89 groups showed cell spreading and F-actin organization degrees between control and CGRP groups or closer to controls, indicating that CGRP’s positive morphological effects also depend on its receptor and PKA pathway activation. These morphological changes, characterized by enhanced cell spreading and improved F-actin organization, suggest that CGRP might influence cell adhesion and migration capacity through cytoskeleton regulation.

#### 3.1.4 CGRP effects on BMSC apoptosis

**FIGURE 3:**
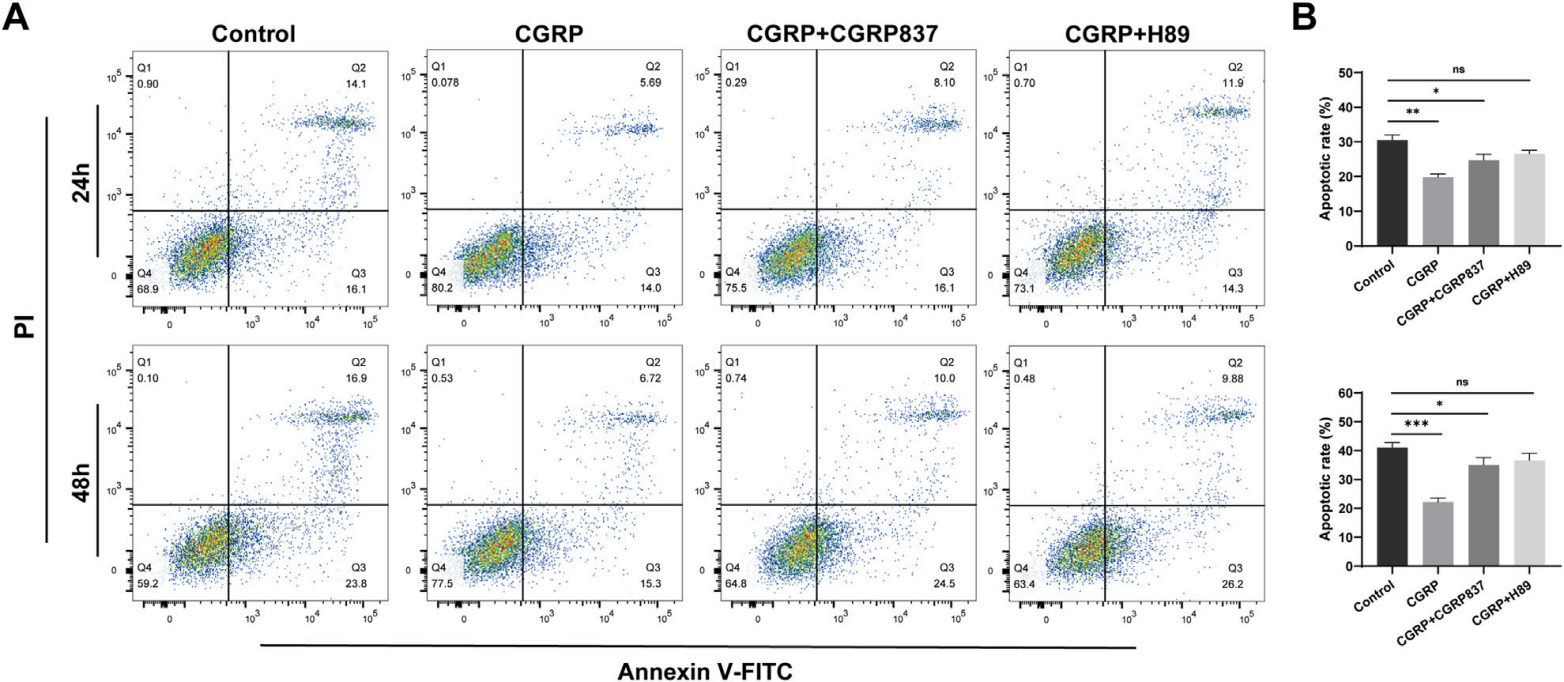
CGRP protects BMSCs from serum deprivation-induced apoptosis: **(A)** representative flow cytometric dot plots showing Annexin V-FITC/PI double staining after 24 and 48 h of serum-free culture. Four distinct cell populations are displayed: viable cells (Annexin V^−^/PI^−^, lower left quadrant), early apoptotic cells (Annexin V^+^/PI^−^, lower right quadrant), late apoptotic cells (Annexin V^+^/PI^+^, upper right quadrant), and necrotic cells (Annexin V^−^/PI^+^, upper left quadrant). Representative plots show control, CGRP (10^−8^ M), CGRP + CGRP8-37 (10^−8^ M CGRP + 10^−6^ M CGRP8-37), and CGRP + H89 (10^−8^ M CGRP + 0.2 μg/mL H89) treatment groups. **(B)** Quantitative analysis of the total apoptotic rate calculated as the sum of early apoptotic (Annexin V^+^/PI^−^) and late apoptotic (Annexin V^+^/PI^+^) cell percentages. CGRP treatment significantly reduced total apoptosis compared to control. CGRP8-37 and H89 co-treatments abolished CGRP’s protective effects, with apoptosis rates returning to control levels. Data represent mean ± SD from independent experiments (n = 6). *P < 0.05 compared to the control group; *P < 0.05 compared to the CGRP group using one-way ANOVA followed by Tukey’s *post hoc* test.

To study CGRP’s protective effects on BMSCs under stress conditions, serum-free culture was used to induce apoptosis. Annexin V-FITC/PI flow cytometry results (Figure 3) showed obvious apoptosis in control group cells after 24 h of serum-free culture. Compared to controls, 10^−8^ M CGRP treatment significantly reduced total BMSC apoptosis rates (calculated as the sum of early and late apoptotic cells: 41.07% ± 1.78% vs. 22.18% ± 1.42%, P < 0.05). However, when CGRP was co-treated with the CGRP receptor antagonist CGRP8-37 or the PKA inhibitor H89, CGRP’s antiapoptotic effects were significantly weakened or reversed, with apoptosis rates approaching control levels (P < 0.05 vs. CGRP group). These results indicate that CGRP can effectively inhibit BMSC apoptosis under stress conditions, with this protective effect depending on CGRP receptor activation and downstream PKA signaling pathway integrity. This suggests that CGRP not only promotes cell proliferation but also enhances cell survival in adverse environments.

#### 3.1.5 CGRP effects on BMSC migration ability

To assess CGRP’s regulatory effects on BMSC migration ability, in this study, we used two complementary methods: scratch assays and Transwell migration assays.

**FIGURE 4:**
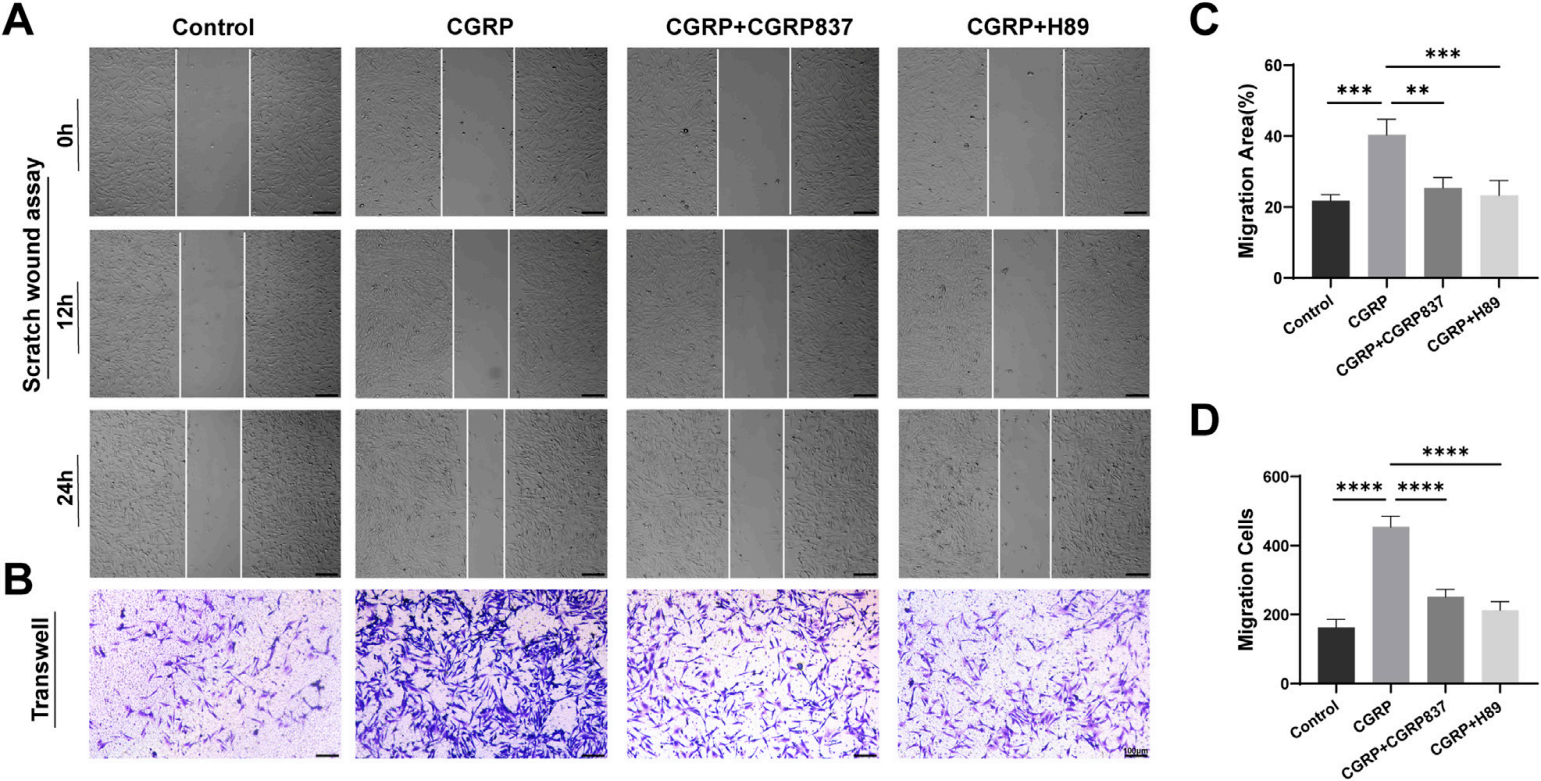
CGRP promotes BMSC migration capacity: **(A, C)** scratch wound healing assay showing accelerated gap closure with CGRP treatment at 0, 12, and 24 h (scale bars: 100 μm). Quantitative analysis of the remaining wound area percentage. **(B, D)** Transwell migration assay demonstrating enhanced chemotactic migration. Representative images of migrated cells stained with crystal violet and quantitative cell counts (scale bars: 100 μm). Data represent mean ± SD. *P < 0.05.

Scratch assay results (Figures 4A,C) showed that at 12 and 24 h post-scratching, CGRP-treated groups had significantly faster scratch healing rates than controls, with significantly reduced scratch areas (21.75 ± 1.78 vs. 40.35 ± 4.42, P < 0.05). CGRP8-37 and H89 could effectively slow CGRP-induced scratch healing processes (P < 0.05 vs. CGRP group).

Transwell migration assay results (Figures 4B,D) further confirmed CGRP’s pro-migration effects from chemotactic migration perspectives. Compared to controls, CGRP significantly promoted BMSC migration toward the bottom of the upper chamber micropore membranes, with obviously increased migrated cell numbers (162 ± 24 vs. 454 ± 31, P < 0.05). Both CGRP8-37 and H89 could significantly inhibit CGRP-induced BMSC migration (P < 0.05 vs. CGRP group), restoring migrated cell numbers to near-control levels.

These two experimental results collectively demonstrate that CGRP has significant chemotactic effects, effectively promoting the directional migration and lateral movement of BMSCs. Effective BMSC migration is a key step for bone tissue to recruit sufficient seed cells to injury sites during damage repair, crucial for initiating and maintaining repair processes. CGRP’s pro-migration effects similarly depend on specific receptor activation and are completely mediated by the downstream PKA signaling pathway.

#### 3.1.6 CGRP effects on BMSC osteogenic differentiation

**FIGURE 5:**
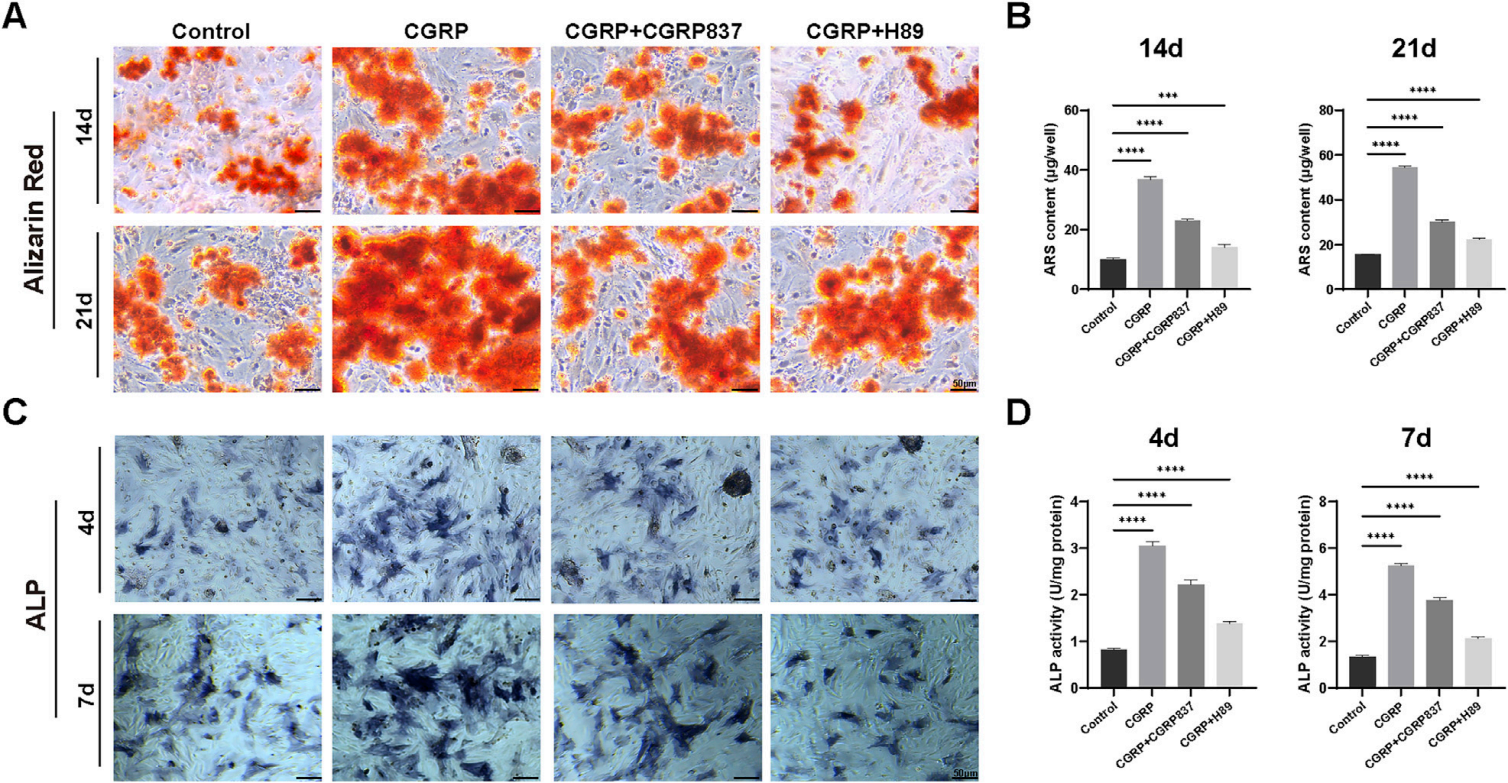
CGRP enhances BMSC osteogenic differentiation: **(A)** Alizarin Red S staining showing calcium nodule formation at days 14 and 21 of osteogenic induction. CGRP treatment resulted in more numerous and deeper-stained orange-red calcified nodules compared to controls. CGRP8-37 and H89 treatments significantly reduced CGRP-induced mineralization enhancement (scale bars: 50 μm). **(B)** Quantitative analysis of Alizarin Red staining using the cetylpyridinium chloride (CPC) extraction method. CGRP treatment showed 3.7-fold and 3.5-fold increases in mineralization at 14 and 21 days, respectively, compared to controls. Both CGRP8-37 and H89 treatments effectively blocked CGRP-induced mineralization enhancement. **(C)** Alkaline phosphatase (ALP) staining demonstrating enhanced early osteogenic marker expression at days 4 and 7. Purple precipitates indicate ALP activity (scale bars: 50 μm). CGRP8-37 and H89 treatments inhibited CGRP-induced osteogenic enhancement. **(D)** Quantitative ALP activity measurements normalized to total protein content. CGRP treatment demonstrated 3.7-fold and 3.9-fold increases in ALP activity at 4 and 7 days, respectively, compared to controls. CGRP + CGRP8-37 and CGRP + H89 groups showed significantly reduced ALP activity compared to CGRP alone, confirming that both receptor blockade and PKA pathway inhibition prevent CGRP’s osteogenic enhancing effects.

Alizarin Red S staining was used to detect the formation of calcified nodules in the extracellular matrix during late-stage osteogenic differentiation. After 14 and 21 days of osteogenic induction culture, results (Figure 5A) showed that CGRP-treated BMSCs formed more numerous, larger, and deeper-stained orange-red calcified nodules, indicating significantly higher mineralization degrees than controls. Quantitative analysis of extracted Alizarin Red dye confirmed these observations, with CGRP treatment showing 3.7-fold and 3.5-fold increases in mineralization at 14 and 21 days, respectively, compared to controls (Figure 5B, P < 0.001). The inhibitor experiments provided essential mechanistic validation of CGRP’s pathway dependence. CGRP + CGRP8-37 treatment significantly reduced mineralization compared to CGRP alone, demonstrating that CGRP receptor antagonism prevents the mineralization-promoting effects. Similarly, CGRP + H89 treatment resulted in markedly decreased calcium deposition, confirming that PKA pathway inhibition blocks CGRP’s ability to enhance matrix mineralization. These findings provide definitive evidence that CGRP’s promotion of osteogenic differentiation requires both specific receptor binding and functional PKA pathway activation.

ALP staining results (Figure 5C) showed that after 4 and 7 days of osteogenic induction culture, compared to controls, CGRP-treated BMSCs showed deeper and more extensive ALP-positive staining (purple-blue precipitates), indicating significantly enhanced ALP activity. Quantitative enzymatic assays corroborated these findings, demonstrating 3.7-fold and 3.9-fold increases in ALP activity at 4 and 7 days, respectively, in CGRP-treated groups compared to controls (Figure 5D, P < 0.001). Similarly, CGRP8-37 and H89 treatments effectively inhibited CGRP-induced ALP enhancement effects. In particular, the CGRP + CGRP8-37 group showed significantly reduced ALP activity compared to CGRP alone, demonstrating that CGRP receptor blockade prevents the osteogenic enhancing effects. The CGRP + H89 group similarly showed diminished ALP activity, confirming that PKA pathway inhibition blocks CGRP’s downstream effects on early osteogenic differentiation. These inhibitor results provide critical mechanistic validation that CGRP’s osteogenic effects require both receptor activation and intact PKA signaling.

These results demonstrate that CGRP can significantly enhance BMSC osteogenic potential and osteoblast-like functional activities, including early ALP activity enhancement and late matrix mineralization. Although these findings indicate CGRP’s capacity to promote osteogenic lineage commitment and functional maturation of BMSCs, further studies would be needed to comprehensively characterize the complete differentiation process and assess the stability of lineage commitment.

#### 3.1.7 Mechanistic studies of CGRP regulation of BMSC osteogenic differentiation through the cAMP/PKA/CREB pathway

**FIGURE 6:**
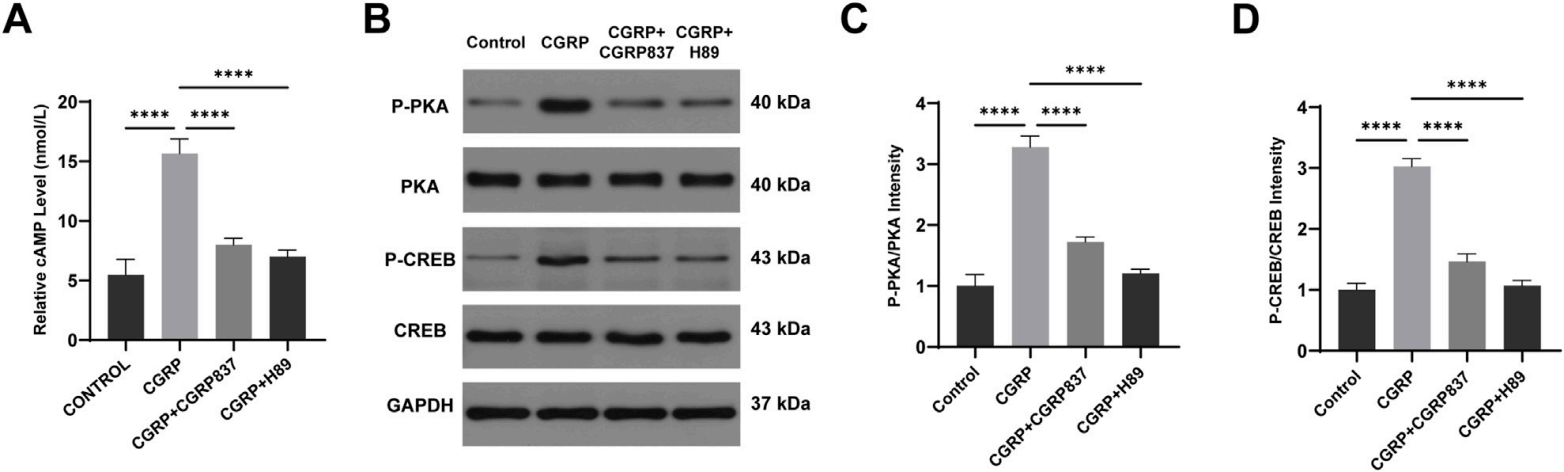
CGRP activates the cAMP/PKA signaling pathway in BMSCs: **(A)** ELISA quantification of intracellular cAMP levels 30 min post-treatment. CGRP rapidly increased cAMP concentration, blocked by CGRP8-37 and H89. Data represent mean ± SD (n = 6). **(B-D)** Western blot analysis of PKA and CREB phosphorylation at 30 min. Representative blots and quantitative analysis of p-PKA/PKA and p-CREB/CREB ratios. Data represent mean ± SD. *P < 0.05.

To investigate CGRP signal transduction initiation steps, intracellular second messenger cAMP concentrations were detected. ELISA results (Figure 6A) showed that after 30 min of CGRP treatment of BMSCs, intracellular cAMP concentrations were significantly elevated compared to controls (P < 0.05). CGRP receptor antagonist CGRP8-37 and PKA inhibitor H89 could block or indirectly block CGRP-induced cAMP level increases, restoring cAMP concentrations to control levels. This result clearly demonstrates that CGRP activates adenylyl cyclase through its receptor, leading to rapid increases in intracellular cAMP levels, which represents the initiating event of cAMP/PKA/CREB signaling pathway activation.

**FIGURE 7:**
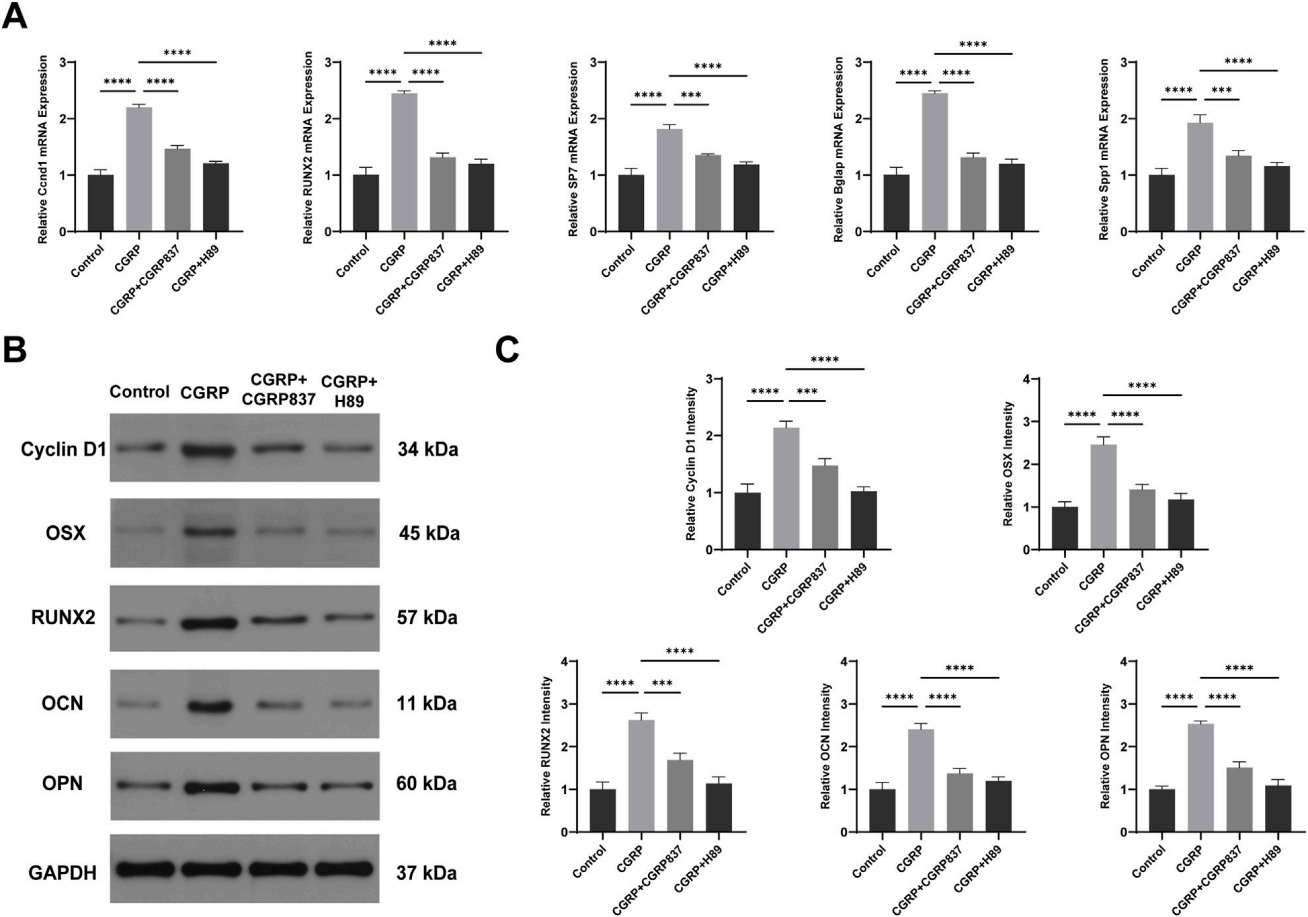
CGRP upregulates osteogenic gene and protein expression *via* the cAMP/PKA/CREB pathway: **(A)** qRT-PCR analysis of osteogenic transcription factors (Runx2 and Osterix) and marker genes (OPN and OCN), along with cell cycle gene (CyclinD1), after 7 days of osteogenic induction. Data normalized to GAPDH and expressed relative to control. **(B, C)** Western blot analysis and quantification of osteogenic proteins and CyclinD1 expression. Data represent mean ± SD. *P < 0.05.

qRT-PCR results (Figure 7A) showed that after 7 days of osteogenic induction culture, CGRP treatment significantly upregulated mRNA expression levels of key osteogenic transcription factors *Runx2* and *Sp7* (Osx), along with late osteogenic marker genes *Spp1* (OPN) and *Bglap* (OCN) in BMSCs (P < 0.05 vs. controls). Meanwhile, cell proliferation and CREB pathway downstream target-related CyclinD1 gene expression were also significantly upregulated (P < 0.05). Both CGRP8-37 and H89 could significantly inhibit CGRP-induced upregulation of these gene expressions (P < 0.05 vs. CGRP group), restoring expression levels close to or back to control levels. These data indicate that CGRP regulates multiple key osteogenic genes and cell cycle-related gene expression at transcriptional levels through its receptor and PKA pathway, providing molecular basis for subsequent protein expression and cellular functional changes.

Western blot analysis results (Figures 6B, 7B–D) further verified signaling pathway activation and downstream effects at protein levels. After CGRP treatment of BMSCs (30 min for phosphorylated protein detection and 7 days for total protein detection), both PKA phosphorylation levels (p-PKA/Total PKA ratios) and its downstream key substrate CREB phosphorylation levels (p-CREB/total CREB ratios) were significantly elevated (P < 0.05 vs. controls). Correspondingly, after 7 days of culture, osteogenesis-related proteins Runx2, Osx, OPN, OCN, and cell cycle protein CyclinD1 expression levels were all significantly upregulated (P < 0.05). Both CGRP8-37 and H89 could significantly weaken CGRP-induced PKA and CREB phosphorylation while inhibiting CGRP-induced upregulation of downstream Runx2, Osx, OPN, OCN, and CyclinD1 protein expressions (P < 0.05 vs. CGRP group). This protein-level evidence demonstrates that CGRP activates the cAMP/PKA/CREB signaling pathway, subsequently upregulating a series of osteogenic differentiation and cell proliferation-related protein expressions, thereby exerting biological effects. *In vitro* experimental results form a complete evidence chain, confirming that CGRP promotes BMSC osteogenic differentiation through cAMP/PKA/CREB pathway molecular mechanisms.

### 3.2 CGRP *in vivo* promotion of rat distraction osteogenesis

#### 3.2.1 Gross observation and radiological assessment

**FIGURE 8:**
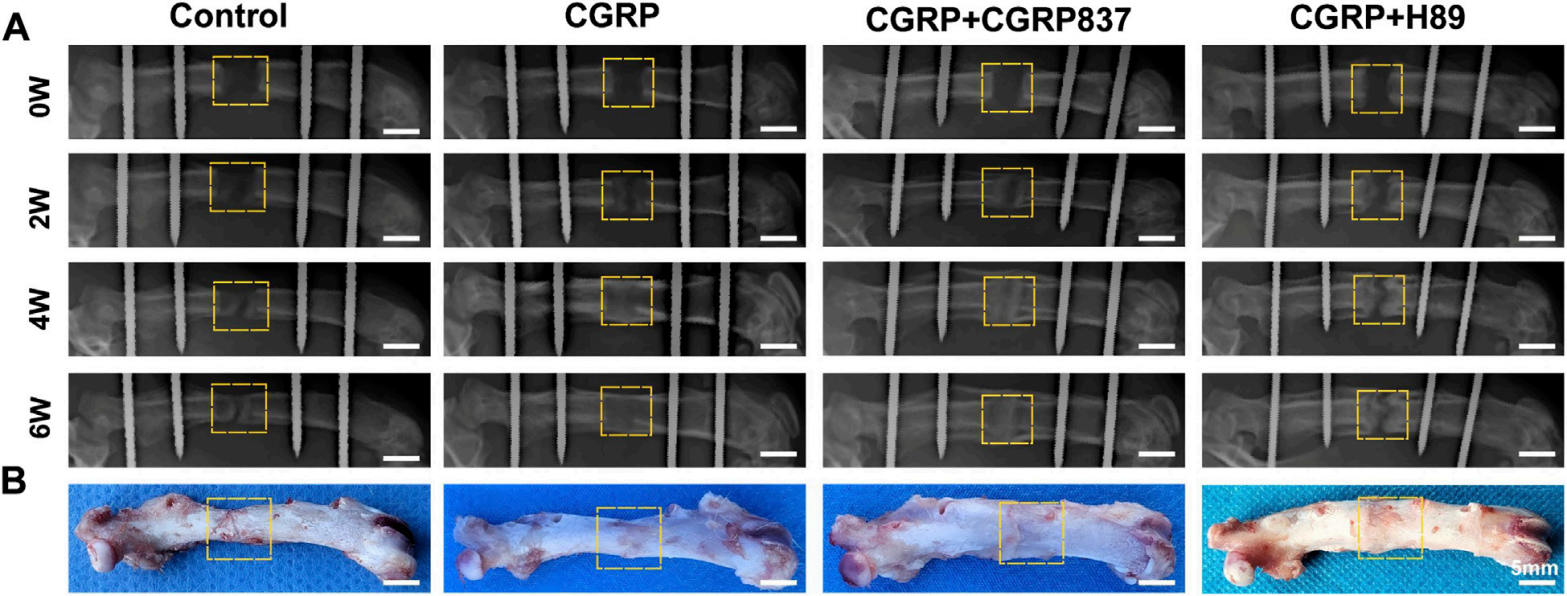
CGRP accelerates bone formation in the rat femoral distraction osteogenesis model: **(A)** serial radiographic images at 2, 4, and 6 weeks post-consolidation showing progressive bone callus formation and maturation. The CGRP group demonstrates superior bone bridging and density. **(B)** Gross morphology of femoral specimens at 6 weeks of consolidation. CGRP treatment resulted in robust bone union with continuous cortical bridging, whereas controls showed incomplete healing with visible gaps.

Gross morphological examination (Figure 8B, showing representative specimens at 6 weeks of consolidation) revealed significant differences in distraction gap healing degrees among groups at consolidation completion. In the control group, distraction gaps mostly showed obvious depressions or only minimal fibrous tissue connections, with inadequate new bone filling that failed to form solid, continuous bone bridges, and some specimens even exhibited pseudarthrosis-like changes. In contrast, in the CGRP treatment group, distraction gaps were almost completely filled with hard new bone tissue, forming relatively robust and continuous bone bridge structures with relatively smooth surfaces and colors approaching normal bone tissue, achieving tight fusion with surrounding host bone and demonstrating excellent bone healing quality and morphology. CGRP + CGRP8-37 and CGRP + H89 groups showed significantly inferior bone healing compared to CGRP alone, with generally larger distraction gaps, insufficient new bone filling, poor or absent bone connections, and gross morphology closer to controls, indicating significant inhibition of CGRP’s healing-promoting effects by the receptor antagonist or PKA inhibitor. These gross morphological differences observed at 6 weeks intuitively reflected different treatment effects on final bone healing outcomes.

Radiological dynamic observation results (Figure 8A) were consistent with gross observation trends. At 2 weeks of consolidation, control groups showed only minimal blurred bone callus shadows within distraction gaps. CGRP groups showed relatively more abundant new bone callus shadows with higher density than controls, with distraction gaps beginning to show blurring tendencies. CGRP + CGRP8-37 and CGRP + H89 groups showed bone callus formation similar to or slightly better than controls but obviously inferior to CGRP groups. Entering 4 weeks of consolidation, CGRP groups showed dramatically increased bone callus amounts with obviously increased density, with most distraction gaps effectively filled by new bone and initial bone continuity formation. Control, CGRP + CGRP8-37, and CGRP + H89 groups still showed relatively low bone callus amounts and density. At 6 weeks of consolidation completion, in the CGRP group, distraction gaps were basically filled with mature, well-bridged bone callus, with high new bone density, relatively clear trabecular structures, and some degrees of medullary recanalization signs, demonstrating excellent bone healing effects. In contrast, in the control, CGRP + CGRP8-37, and CGRP + H89 groups, distraction gaps still failed to achieve complete healing, with X-ray images showing partial areas with fibrous connections or only minimal immature bone callus formation. These results preliminarily indicate that CGRP can significantly promote distraction osteogenesis, with this effect being blocked by the CGRP receptor antagonist and the PKA inhibitor.

#### 3.2.2 Micro-CT assessment of new bone quality and quantity

**FIGURE 9:**
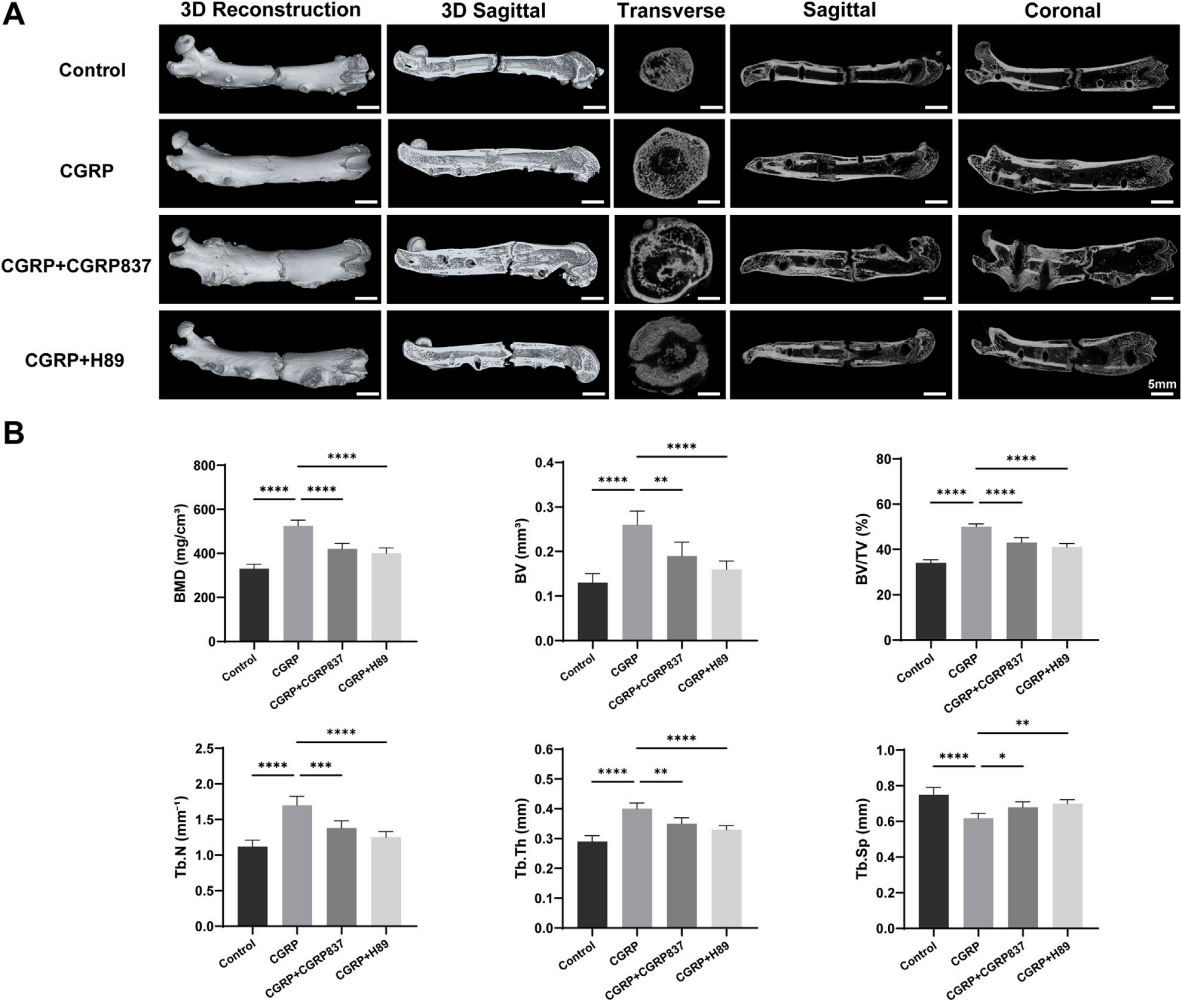
**(A)** Micro-CT analysis reveals enhanced bone microarchitecture with CGRP treatment: three-dimensional reconstructed images and quantitative analysis of distraction gaps at 6 weeks of consolidation. **(B)** CGRP significantly improved bone mineral density (BMD), bone volume fraction (BV/TV), trabecular number (Tb.N), and trabecular thickness (Tb.Th) while reducing trabecular separation (Tb.Sp). Data represent mean ± SD. *P < 0.05.

After 6 weeks of consolidation completion, micro-CT three-dimensional reconstruction and quantitative analysis of new bone in rat femoral distraction areas were performed (Figure 9). Three-dimensional reconstruction images intuitively showed that CGRP group distraction areas had the most abundant new bone amounts, with dense and well-connected trabecular structures forming relatively complete and solid bone bridges, with better medullary recanalization degrees. In contrast, in the control, CGRP + CGRP8-37, and CGRP + H89 groups, distraction area bone defects remained relatively obvious, with sparse new bone amounts and poor bone continuity.

BMD values in the CGRP group were significantly higher than those in the control, CGRP + CGRP8-37, and CGRP + H89 groups (P < 0.05). In CGRP + CGRP8-37 and CGRP + H89 groups, BMD values showed no significant differences from those in controls but were significantly lower than those in the CGRP group (P < 0.05). In the CGRP group, BV/TV values were similarly significantly higher than those in all other three groups (P < 0.05). In the CGRP + CGRP8-37 and CGRP + H89 groups, BV/TV values were similar to those in controls but significantly lower than those in the CGRP group (P < 0.05). Tb.N and Tb.Th values in the CGRP group were both significantly higher than those in all other groups (P < 0.05). In the CGRP + CGRP8-37 and CGRP + H89 groups, Tb.N and Tb.Th values showed no significant improvements compared to those in controls but were significantly lower than those in the CGRP group (P < 0.05). In the CGRP group, Tb. Sp values were significantly lower than those in all other groups (P < 0.05), indicating a more compact new bone structure with smaller trabecular spaces. In the CGRP + CGRP8-37 and CGRP + H89 groups, Tb. Sp values were similar to those in controls but significantly higher than those in the CGRP group (P < 0.05).

These comprehensive micro-CT assessment results from three-dimensional structure and bone mass perspectives further powerfully confirmed that CGRP can significantly promote high-quality new bone formation and effective mineralization during distraction osteogenesis *in vivo*, with this promoting effect depending on CGRP receptor and PKA pathway activation.

#### 3.2.3 New bone biomechanical performance assessment

**FIGURE 10:**
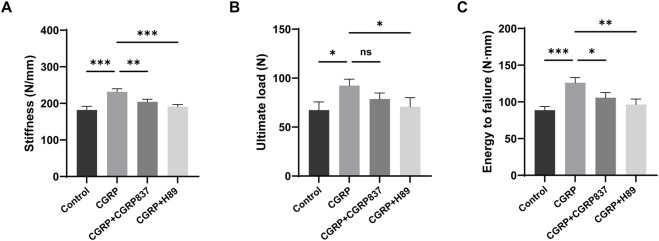
CGRP enhances biomechanical properties of regenerated bone: three-point bending test results at 6 weeks of consolidation showing **(A)** ultimate load, **(B)** energy to failure, and **(C)** stiffness measurements. CGRP treatment significantly improved all mechanical parameters compared to controls. CGRP8-37 and H89 abolished these beneficial effects. Data represent mean ± SD. *P < 0.05.

After 6 weeks of consolidation completion, three-point bending tests were performed on regenerated femurs from each group to assess mechanical strength (Figure 10). The regenerated femurs in the CGRP group could withstand significantly higher ultimate loads than those in the control, CGRP + CGRP8-37, and CGRP + H89 groups (P < 0.05). In the CGRP + CGRP8-37 and CGRP + H89 groups, ultimate loads showed no significant differences from those in controls but were significantly lower than those in the CGRP group (P < 0.05). In the CGRP group, energy to failure was similarly significantly higher than that in the other three groups (P < 0.05), indicating that new bone could absorb more energy before fracture with better toughness. In the CGRP + CGRP8-37 and CGRP + H89 groups, energy to failure was similar to that in controls but significantly lower than that in the CGRP group (P < 0.05). Stiffness values in the CGRP group were also significantly higher than those in the other three groups (P < 0.05). In the CGRP + CGRP8-37 and CGRP + H89 groups, stiffness showed no significant differences from that in controls but it was significantly lower than that in the CGRP group (P < 0.05).

These biomechanical test results fully demonstrate that local CGRP application can not only effectively increase new bone quantity and improve microstructure but, more importantly, can also significantly enhance biomechanical performance with significant improvements in strength, stiffness, and toughness. CGRP receptor antagonist and PKA inhibitor application completely eliminated CGRP’s mechanical enhancement effects, further confirming the critical role of the CGRP signaling pathway in regulating bone regeneration functional recovery.

#### 3.2.4 New bone histological and immunohistochemical assessment

**FIGURE 11:**
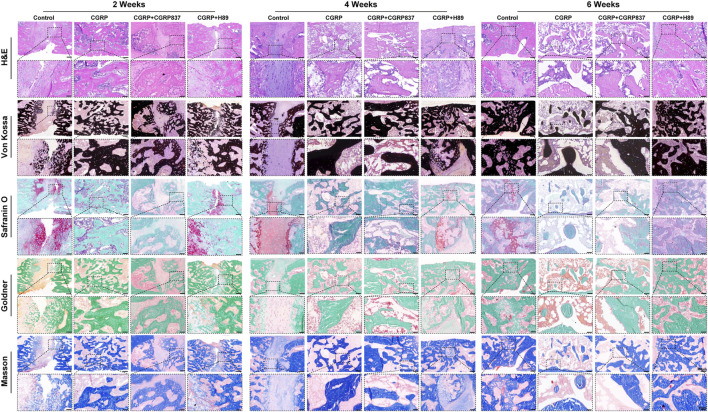
Histological analysis demonstrates superior bone quality with CGRP treatment: representative sections at 2, 4, and 6 weeks of consolidation stained with Von Kossa (mineralized bone, black), Masson trichrome (collagen fibers, blue; bone, red), Goldner trichrome (mineralized bone, green; osteoid, red), and safranin O–fast green (cartilage, red; bone, green). The CGRP group shows extensive mineralized bone formation with mature lamellar structure and minimal residual cartilage (scale bars: 200 μm and 50 μm).

Histological staining results (Figure 11) showed significant differences in new bone quality and quantity among groups. Through Von Kossa staining observing mineralized bone distribution, the distraction areas in the CGRP group showed abundant, dense black mineralized deposits forming continuous and robust bone bridge structures, with relatively mature trabecular morphology and relatively regular arrangements. In contrast, in the control, CGRP + CGRP8-37, and CGRP + H89 groups, distraction areas showed only minimal, scattered, and irregularly distributed mineralized deposits, with most areas still occupied by unmineralized fibrous or cartilaginous tissues and poor bone connections.

Masson trichrome and Goldner trichrome staining further revealed new bone maturity and matrix components. In CGRP groups, new bone tissues were more mature, with abundant orderly arranged collagen fibers (Masson blue and Goldner green), evenly distributed osteocytes, and relatively minimal osteoid matrix (Goldner red), indicating active bone remodeling and good ossification. Other three groups showed abundant fibrous tissues and minimal immature osteoid matrix, forming sharp contrasts, with sparse, thin, and disorderly arranged trabeculae.

Through Safranin O–Fast Green staining observing cartilage components, minimal cartilaginous endochondral ossification signs might exist in distraction areas at early experimental time points. However, by 6 weeks of consolidation completion, the distraction areas in the CGRP group showed almost no remaining cartilage tissue (safranin O red portions), mainly replaced by mature lamellar bone structures, indicating high bone remodeling processes and tissue maturity. In contrast, other three groups might still have partial fibrocartilaginous tissues or incompletely absorbed cartilage components. These histological results clearly confirmed from morphological perspectives that CGRP can significantly promote new bone quality and quantity improvements in the distraction area, whereas CGRP receptor antagonist and PKA inhibitor application severely hindered these beneficial processes.

**FIGURE 12:**
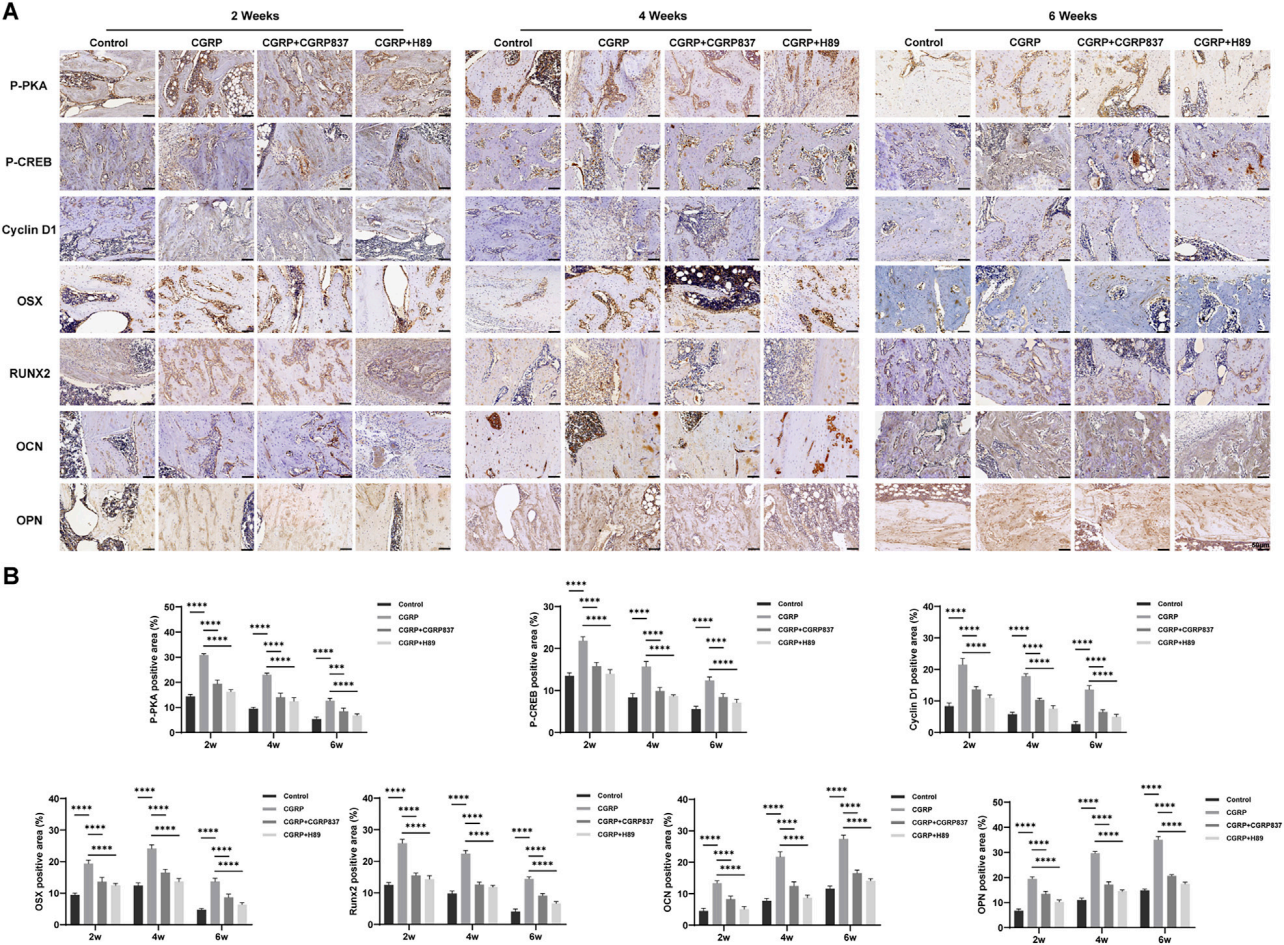
Immunohistochemical analysis confirms activation of osteogenicmarkers and the signaling pathway: **(A)** representative immunohistochemical staining and quantitative analysis of osteogenic transcription factors (Runx2 and Osterix), bone matrix proteins (OPN and OCN), and pathway molecules (p-PKA and p-CREB) in distraction gap tissues at 6 weeks of consolidation. Brown staining indicates positive expression. **(B)** CGRP treatment significantly enhanced all markers (scale bars: 50 μm). Data represent mean ± SD. *P < 0.05.

Immunohistochemical staining results (Figure 12) showed that in the consolidation-period distraction areas, the new bone tissues of the CGRP group (especially in active osteoblasts and young osteocytes) showed significantly higher positive expression levels of Runx2, Osx, OPN, and OCN than those in control, CGRP + CGRP8-37, and CGRP + H89 groups (P < 0.05). In the CGRP + CGRP8-37 and CGRP + H89 groups, osteogenic marker expression levels were similar to or slightly higher than those in controls but significantly lower than those in the CGRP group (P < 0.05). New bone areas in the CGRP group showed significantly higher p-PKA- and p-CREB-positive staining levels than those in the control, CGRP + CGRP8-37, and CGRP + H89 groups (P < 0.05). In the CGRP + CGRP8-37 and CGRP + H89 groups, p-PKA- and p-CREB-positive staining levels were significantly lower than those in the CGRP group, approaching control levels (P < 0.05).

This series of immunohistochemical results, highly consistent with *in vitro* experimental results, indicates that CGRP can similarly activate the cAMP/PKA/CREB signaling pathways in local bone tissues *in vivo*, upregulating key osteogenic transcription factors and bone matrix protein expression, thereby promoting high-quality new bone formation during distraction osteogenesis. These findings provide direct histological and molecular evidence for CGRP exerting bone regeneration-promoting effects through this signaling pathway *in vivo*.

## 4 Discussion

Large bone defect repair remains a major challenge in clinical orthopedics, whereas DO technology provides a promising solution, but prolonged consolidation periods and potential complications limit its clinical benefits (Schmidmaier and Moghaddam, 2015; Papakostidis et al., 2013). In this study, we systematically investigated neuropeptide CGRP effects on BMSC osteogenic differentiation *in vitro* and its role in promoting bone regeneration in rat femoral DO models *in vivo*, revealing the critical role of the cAMP/PKA/CREB signaling pathway. Our results demonstrate that CGRP can significantly enhance BMSC proliferation, migration, antiapoptotic capacity, and osteogenic differentiation, effectively promoting DO new bone formation, mineralization, and mechanical property improvements *in vivo*, with all beneficial effects depending on cAMP/PKA/CREB signaling pathway activation.

*In vitro* experiments first confirmed 10^−8^ M CGRP as optimal concentration for promoting BMSC proliferation and established that CGRP effectively inhibits serum-free culture-induced apoptosis, enhancing cell survival capacity. This is consistent with previous reports of CGRP-protective effects on various cell types (Wu et al., 2018; Mrak et al., 2010). Both scratch and Transwell assays confirmed that CGRP significantly promotes BMSC migration ability, suggesting that it might provide more abundant seed cells for new bone formation by increasing BMSC numbers and recruitment in the DO area.

Regarding BMSC lineage commitment, the primary focus of our study was osteogenic differentiation, where CGRP significantly enhanced ALP activity and calcified nodule formation while upregulating key osteogenic transcription factors Runx2 and Osterix, along with late osteogenic markers OPN and OCN (Zeng et al., 2024; Tang et al., 2011; Kuzan et al., 2024). Although we demonstrated CGRP’s capacity to promote osteogenic lineage development, the effects on alternative differentiation pathways warrant consideration. Previous studies have suggested that CGRP may influence the balance between osteogenic and adipogenic differentiation, with some evidence indicating that CGRP treatment can suppress adipogenesis while promoting osteogenesis in MSCs, potentially through modulation of key transcription factors such as PPARγ and C/EBPα that regulate adipocyte differentiation. Regarding chondrogenic differentiation, limited evidence exists for direct CGRP effects on cartilage formation although the cAMP/PKA signaling pathway we identified has been implicated in chondrocyte differentiation regulation. Future studies examining CGRP’s effects on multiple lineage commitments simultaneously would provide a more comprehensive understanding of its role in MSC fate determination.

In this study, we investigated molecular mechanisms underlying the biological effects of CGRP. We found that CGRP treatment rapidly increased the intracellular cAMP levels of BMSCs while significantly promoting PKA phosphorylation activation and its downstream substrate CREB phosphorylation. CREB, as a key transcription factor, regulates multiple cell proliferation, survival, and differentiation-related gene expressions after phosphorylation activation, including CyclinD1 detected in this study (Molnar et al., 2014; Yan et al., 2013). CyclinD1 is a key cell cycle-regulatory protein, whose upregulation promotes cell proliferation (Daniel et al., 2014). Using the CGRP-specific receptor antagonist CGRP8-37 and the PKA-specific inhibitor H89, we found that CGRP-induced BMSC proliferation, anti-apoptosis, migration, osteogenic differentiation, and related signaling molecule activation and gene expression were all significantly inhibited. These results demonstrate the central role of the cAMP/PKA/CREB signaling pathway in the biological function regulation of CGRP-mediated BMSCs. Previous studies also suggested that CGRP might exert effects through activating cAMP/PKA pathways in other cell types (Holzmann, 2013; García-Morales et al., 2017), whereas in this study, the key position of this pathway in CGRP-regulated osteogenic differentiation of BMSCs was systematically confirmed for the first time (Vandamme et al., 2012; Inoue et al., 2013).

*In vivo* DO models validated CGRP bone regeneration-promoting effects. Through comprehensive assessments including gross observation, serial radiological evaluation, micro-CT analysis, biomechanical testing, and histological and immunohistochemical analyses, we evaluated CGRP effects on DO new bone. Results showed that local CGRP injection significantly accelerated new bone formation and mineralization and improved bone mass (BMD and BV/TV) and microstructure, ultimately significantly enhancing new bone biomechanical strength. These results align with previous reports of CGRP promoting fracture healing and bone regeneration (Xu et al., 2020; Mi et al., 2021; Zhang et al., 2016; Zhao et al., 2024; Chen et al., 2021). This study’s innovation lies in applying CGRP to DO models while systematically evaluating comprehensive effects on new bone quality and mechanical performance.

More importantly, *in vivo* experiments similarly confirmed cAMP/PKA/CREB signaling pathway criticality in CGRP bone regeneration promotion. In distraction area new bone tissues of the CGRP treatment group, we observed significantly elevated p-PKA and p-CREB expression levels, accompanied by upregulated expressions of the osteogenic markers Runx2, Osterix, OPN, and OCN. CGRP receptor antagonist CGRP8-37 or PKA inhibitor H89 application significantly weakened CGRP bone regeneration-promoting effects and related signaling molecule activation and osteogenic marker expression. This is highly consistent with *in vitro* experimental results, collectively revealing that CGRP activates its receptor, upregulates intracellular cAMP levels, subsequently activates PKA, and phosphorylates CREB, ultimately promoting a series of osteogenesis-related gene expressions, thereby exerting powerful bone formation-promoting effects both *in vitro* and *in vivo*. These results demonstrate that the cAMP/PKA/CREB signaling pathway plays a significant role in mediating CGRP’s effects on BMSCs, as evidenced by rapid cAMP elevation, PKA phosphorylation, and subsequent CREB activation leading to upregulation of osteogenic and proliferation-related proteins. However, it is important to recognize that osteogenic differentiation involves complex, interconnected signaling networks, and CGRP’s effects likely involve crosstalk with multiple pathways beyond cAMP/PKA/CREB signaling. Previous studies have demonstrated that osteogenic differentiation requires coordinated activation of various signaling cascades, including Wnt/β-catenin, BMP/Smad, MAPK, and Notch pathways, among others. Although our use of specific inhibitors (CGRP8-37 and H89) provides strong evidence for cAMP/PKA/CREB involvement, the residual biological activity observed in some assays suggests that additional mechanisms may contribute to CGRP’s overall effects. Furthermore, the cAMP/PKA/CREB pathway itself exhibits extensive crosstalk with other signaling networks, potentially amplifying or modulating CGRP’s actions through secondary pathway activation. Future studies utilizing pathway-specific inhibitors or transcriptomic analyses would help elucidate the full spectrum of molecular mechanisms underlying CGRP’s osteogenic effects.

Although the cAMP/PKA/CREB signaling pathway was primarily focused on in our study, it is important to acknowledge that CGRP’s osteogenic effects likely involve a broader signaling context through integration with additional pathways. Recent evidence demonstrates that CGRP also activates mitogen-activated protein kinase (MAPK) signaling cascades, particularly ERK1/2 and p38 MAPK pathways, to promote osteogenic differentiation of mesenchymal stem cells (Jiang et al., 2022). Studies have shown that CGRP treatment rapidly induces phosphorylation of both ERK1/2 and p38 MAPK in a time-dependent manner, with peak activation occurring within 30 min of stimulation, and that pharmacological inhibition of these pathways significantly attenuates CGRP-induced osteogenic marker expression (Jiang et al., 2022). Importantly, the cAMP/PKA/CREB pathway we identified appears to function synergistically with MAPK signaling networks rather than in isolation. Previous research has demonstrated crosstalk between cAMP and MAPK signaling, where PKA can activate downstream targets that subsequently influence MAPK cascades (Greenblatt et al., 2013). Additionally, p38 MAPK can phosphorylate and activate CREB, creating a convergence point between MAPK and cAMP signaling that may amplify transcriptional responses to CGRP treatment (Cuenda and Rousseau, 2007). This integrated signaling model provides a more comprehensive framework for understanding how CGRP orchestrates the complex molecular program required for osteogenic differentiation. The coordinated activation of multiple pathways likely ensures robust and sustained upregulation of key osteogenic transcription factors while simultaneously promoting cell survival and migration through complementary mechanisms.

Beyond MAPK signaling, CGRP’s osteogenic effects may also involve other important pathways critical for bone formation. The Wnt/β-catenin pathway, a master regulator of osteoblast differentiation and bone formation, represents another potential target of CGRP action. Although we did not directly investigate Wnt signaling in this study, previous research has demonstrated that neuropeptides can modulate β-catenin stability and nuclear translocation, suggesting possible interactions between CGRP and canonical Wnt signaling (Leucht et al., 2008). Additionally, given CGRP’s well-established vasodilatory properties, angiogenesis-related pathways including VEGF and Notch signaling may contribute to CGRP’s bone regeneration effects by enhancing vascularization of the distraction gap, which is essential for delivering nutrients and osteoprogenitor cells to support new bone formation (Hu and Olsen, 2016). Importantly, our findings do not exclude the involvement of these additional pathways but rather establish the cAMP/PKA/CREB axis as one critical component within a broader signaling network. The robust effects we observed with pathway-specific inhibitors (CGRP8-37 and H89) demonstrate the necessary role of cAMP/PKA/CREB signaling while leaving open the possibility that CGRP simultaneously activates complementary pathways to achieve maximal osteogenic responses. This multifactorial signaling model is consistent with the complex nature of osteogenesis, where multiple pathways converge to ensure robust bone formation under diverse physiological and pathological conditions.

The findings have significant clinical implications for optimizing DO protocols. Current DO treatments often require 6–12 months of consolidation, during which patients face risks of complications, including nonunion, hardware failure, and refracture. CGRP’s ability to enhance bone formation quality and accelerate consolidation could potentially reduce treatment duration and improve patient outcomes. The demonstrated improvements in biomechanical properties are particularly relevant as they suggest enhanced functional recovery and reduced re-fracture risk.

Despite meaningful findings, this study has limitations. First, only SD rat femoral DO models were used; future validation in other animal models (such as large animal models) or different bone defect models is needed. Second, in this study, we mainly focused on the cAMP/PKA/CREB signaling pathway, but CGRP mechanisms might involve other signaling pathways such as MAPK and Wnt/β-catenin pathways, with potentially complex crosstalk warranting further exploration. Additionally, CGRP delivery in this study used local multiple injections; future exploration of more efficient and convenient CGRP sustained-release delivery systems could maintain effective target area concentrations while reducing administration frequency. For example, combining biomaterials to construct CGRP controlled-release systems might better facilitate clinical translation. Finally, this study’s observation endpoints mainly focused on the 6-week consolidation phase; longer-term bone remodeling and its potential effects require further investigation.

The clinical translation of CGRP-based therapies will require sophisticated delivery systems that overcome the peptide’s inherent limitations while providing sustained therapeutic concentrations at target sites. The short half-life of CGRP (6–25 min) and the need for repeated injections demonstrated in our study highlight critical translational challenges that must be addressed for practical clinical implementation. Several promising biomaterial-based approaches that could provide controlled and sustained CGRP release have emerged. Injectable hydrogel systems offer particular promise for minimally invasive delivery, with self-healing nanocomposite hydrogels demonstrating the ability to provide sustained protein release over extended periods while maintaining bioactivity (Zhang et al., 2020). These systems can be precisely delivered to distraction gaps through minimally invasive injection, eliminating the need for repeated surgical access while providing controlled peptide release profiles. Additionally, peptide-functionalized scaffolds represent another advanced strategy, where CGRP could be incorporated into three-dimensional biomimetic structures that not only deliver the neuropeptide but also provide mechanical support for bone regeneration (Azadi et al., 2024). Such integrated approaches would address both the biological (sustained CGRP delivery) and mechanical (structural support) requirements for optimal distraction osteogenesis outcomes. The development of these delivery platforms would also enable combination therapies, where CGRP could be co-delivered with complementary factors such as growth factors or other neuropeptides to achieve synergistic effects. Furthermore, smart delivery systems responsive to local biochemical or mechanical cues could provide temporal control over CGRP release, potentially matching the natural healing phases during distraction osteogenesis. These technological advances are essential for translating the promising experimental findings demonstrated in our study into clinically viable therapeutic strategies that can improve patient outcomes while reducing treatment burden and healthcare costs.

Although promising therapeutic effects of CGRP treatment have been demonstrated in our study, several safety considerations warrant evaluation for clinical translation. The primary concern involves CGRP’s vasodilatory properties, which could theoretically cause systemic hypotensive effects if significant amounts enter circulation. However, our local injection approach and CGRP’s rapid degradation (a half-life of 6–25 min) likely minimize systemic exposure. No adverse effects were observed in treated animals, including no cardiovascular compromise or injection site reactions. Nevertheless, clinical applications would require careful dose optimization and monitoring, particularly in patients with cardiovascular conditions. Additionally, although CGRP is endogenous to bone tissue, potential immunogenic reactions to exogenous administration should be considered. Long-term safety studies examining repeated administration and effects on normal bone remodeling would be valuable for comprehensive assessment.

Future research directions might include the following: (1) developing novel CGRP sustained-release systems such as PLGA microsphere, hydrogel, or nanoparticle-based delivery carriers to achieve sustained, stable CGRP release at bone defect sites, evaluating repair effects in different bone defect models. (2) Deeply investigating synergistic effects and molecular crosstalk mechanisms between CGRP and other osteogenic factors or signaling pathways (such as BMPs, VEGF, and Notch) to further enhance bone regeneration effects through combined applications. (3) Combining genetic engineering technologies, such as using viral vectors, to overexpress CGRP receptors or downstream key molecules in BMSCs to enhance cellular CGRP responsiveness. (4) Further clarifying sensory nerve-specific roles in DO processes and how mechanical distraction forces regulate local CGRP release and expression. (5) Conducting larger-scale preclinical studies to establish more solid foundations for CGRP eventual clinical orthopedic disease treatment applications.

## 5 Conclusion

In summary, in this study, we, through systematic *in vitro* and *in vivo* experiments, first confirmed that CGRP can effectively promote BMSC proliferation, migration, anti-apoptosis, and osteogenic differentiation through activating the cAMP/PKA/CREB signaling pathway, significantly accelerating new bone formation, mineralization, and mechanical property improvements in rat femoral distraction osteogenesis models. These findings not only deepen our understanding of CGRP mechanisms in bone regeneration but also provide important experimental evidence and theoretical foundations for developing CGRP-based bone defect repair strategies, suggesting that CGRP has promising applications as an endogenous bone regeneration-promoting factor.

## Data Availability

The original contributions presented in the study are included in the article/Supplementary Material, further inquiries can be directed to the corresponding author.
